# Hypoxia inducible factor-1α regulates microglial innate immune memory and the pathology of Parkinson’s disease

**DOI:** 10.1186/s12974-024-03070-2

**Published:** 2024-03-30

**Authors:** Hongtian Dong, Xiaoshuang Zhang, Yufei Duan, Yongtao He, Jiayin Zhao, Zishan Wang, Jinghui Wang, Qing Li, Guangchun Fan, Zhaolin Liu, Chenye Shen, Yunhe Zhang, Mei Yu, Jian Fei, Fang Huang

**Affiliations:** 1 Department of Translational Neuroscience, Jing’an District Centre Hospital of Shanghai, State Key Laboratory of Medical Neurobiology and MOE Frontiers Center for Brain Science, Institutes of Brain Science, Fudan University, 138 Yixueyuan Road, Shanghai, 200032 China; 2 School of Life Science and Technology, Tongji University, 1239 Shipping Road, Shanghai, 200092 China; 3 Shanghai Engineering Research Center for Model Organisms, Shanghai Model Organisms Center, INC., Shanghai, 201203 China

**Keywords:** Innate immune memory, Parkinson’s disease, Microglia, Hif-1α

## Abstract

Neuroinflammation is one of the core pathological features of Parkinson’s disease (PD). Innate immune cells play a crucial role in the progression of PD. Microglia, the major innate immune cells in the brain, exhibit innate immune memory effects and are recognized as key regulators of neuroinflammatory responses. Persistent modifications of microglia provoked by the first stimuli are pivotal for innate immune memory, resulting in an enhanced or suppressed immune response to second stimuli, which is known as innate immune training and innate immune tolerance, respectively. In this study, LPS was used to establish in vitro and in vivo models of innate immune memory. Microglia-specific *Hif-1α* knockout mice were further employed to elucidate the regulatory role of HIF-1α in innate immune memory and MPTP-induced PD pathology. Our results showed that different paradigms of LPS could induce innate immune training or tolerance in the nigrostriatal pathway of mice. We found that innate immune tolerance lasting for one month protected the dopaminergic system in PD mice, whereas the effect of innate immune training was limited. Deficiency of HIF-1α in microglia impeded the formation of innate immune memory and exerted protective effects in MPTP-intoxicated mice by suppressing neuroinflammation. Therefore, HIF-1α is essential for microglial innate immune memory and can promote neuroinflammation associated with PD.

**Supplementary Information:**

The online version contains supplementary material available at 10.1186/s12974-024-03070-2.

## Background

Neurodegenerative diseases are characterized by the progressive loss of specific neurons within the central nervous system (CNS) [1]. Parkinson’s disease (PD), the second most common degenerative disorder after Alzheimer's disease (AD), is primarily characterized by the progressive loss of dopaminergic neurons and terminals in the nigrostriatal pathway. This loss is accompanied by motor impairments, as well as various non-motor symptoms. The etiology and pathogenesis of PD are complicated, involving a combination of genetic and environmental factors. Since the first case of PD was reported over two hundred years ago, researchers have carried out extensive studies on its molecular mechanisms, including aggregation of α-synuclein, neuroinflammation, oxidative stress, and mitochondrial dysfunction [2–4]. Microglia, serving as resident macrophages in the CNS, play a critical role in neuroinflammation, which is closely associated with the progression of PD [5, 6].

In the past, immune memory has been viewed as a unique property of the adaptive immune system. However, it is now known that memory traits of innate immunity are widespread among various organisms, from plants, invertebrates to mammals [7–9]. As the primary innate immune cells in the brain, microglia retain memory of the first stimulus and modulate the intensity of immune responses to the second stimulus [10, 11]. The first stimulus can be categorized into two types: ‘priming’, which leads to an enhanced secondary immune response (termed innate immune training, TR) or ‘desensitizing’, which results in a suppressive secondary immune response (termed innate immune tolerance, TL) [12, 13]. However, predicting the neurological outcomes of innate immune responses in neurodegenerative diseases is challenging, as it is influenced by various factors such as the microenvironment within the brain, the progression of diseases and other factors. In a pro-inflammatory environment, numerous inflammatory mediators, including pro-inflammatory cytokines (such as IL-1β, IL-6, TNF-α) and nitric oxide (NO), can exert neurotoxic effects. Conversely, the release of anti-inflammatory cytokines (such as IL-4 and IL-10) and nutritional factors (such as NGF, IGF-1 and BDNF) has been shown to be beneficial for neuroprotection [14–21]. Under conditions different from homeostasis, immunological cells acquiring innate immune memory (IIM) undergo phenotypic, functional, and even metabolic shifts. Several molecules, such as hypoxia-inducible factor-1a (HIF-1α), are involved in mediating these changes [11, 22–24].It is highly likely that modifications in microglia contribute significantly to the influence of IIM on the progression of neurological disorders.

The HIF transcription factor, composed of α and β subunits, was initially discovered for its crucial role in cellular adaptation to hypoxia. Originally, the expression of HIF-1α was considered to be solely influenced by hypoxic conditions. However, growing evidence has shown that inflammation, infection and cancer can also facilitate HIF-1α stabilization [25, 26]. HIF-1α has been found to play a role in the activation and polarization of macrophages, as well as regulating cell metabolism and function through interactions with NF-κB signaling pathways, which induce the expression of inflammatory cytokines such as IL-1β [27–30]. The deficiency of HIF-1α in macrophages has been shown to impair the inflammatory response and potentially interfere with innate immune memory (IIM) [22]. A previous study has demonstrated that microglial HIF-1α is a critical essential mediator for IIM in the brain [12], and the expression level of HIF-1α is elevated in microglia associated with amyloid β plaques in AD mice [31, 32]. Similarly, the upregulation of HIF-1α is associated with the pathological stages in patients with AD and PD [32, 33]. However, the involvement of microglial HIF-1α-mediated IIM in the pathological process of PD remains unclear.

In this study, we utilized the methods previously outlined in the literature for establishing IIM models in microglial BV2 cells and mice to investigate the role of microglial IIM in PD [12, 34]. For the in vivo study, we established acute and chronic innate immune memory (AIIM and CIIM) mouse models. In the AIIM model, the interval between first stimulus (1xLPS for TR, and 3xLPS for TL) and second stimulus (1xLPS) was one day; in the CIIM model, MPTP, the second stimulus, was administered to the mice one month after the first stimulus (1xLPS for TR, and 4xLPS for TL) to establish PD mice with IIM. By analyzing the differentially expressed genes between the TR and TL groups, we verified the regulatory effect of HIF-1α on IIM in microglia. The expression of HIF-1α in microglia was further analyzed in IIM PD mice. Additionally, utilizing microglia-specific *Hif-1α* knockout mice, we explored the role of HIF-1α in IIM and its impact on dopaminergic neuron damage in PD mice.

## Methods

### Animals

Male C57BL/6 mice aged 10–12 weeks and *Hif-1α*^flox/flox^ and *Hif-1α*^flox/flox^; *Tmem119*^CreERT2^ male mice were provided by Shanghai Model Organisms Center, Inc. The mice were housed in the IVC system, which maintained a suitable temperature and circadian rhythm. All the animal experiments were conducted in accordance with the guidelines of the Institutional Animal Care and Use Committee of Fudan University, Shanghai Medical College.

### Drug treatment and establishment of mouse model

#### Acute innate immune memory (AIIM) model

Mice were weighed and then injected intraperitoneally with LPS-1, LPS-2 or LPS-3 (Sigma, USA) at a dose of 500 μg/kg at the same time daily. The mice were intraperitoneally injected with a single dose of LPS (1xLPS, first stimulus for TR) or with three consecutive doses of LPS (3xLPS, first stimulus for TL). The following day, another single dose of LPS (second stimulus) was injected to all the mice to induce AIIM. The control group was injected with normal saline (NS).

#### Chronic innate immune memory (CIIM) model

Mice were weighed and then injected intraperitoneally with LPS at a dose of 500 μg/kg at the same time daily. A single-dose of LPS (1xLPS, serving as the first stimulus for TR) or multiple doses of LPS (4xLPS, serving as the first stimulus for TL) were injected to mice, and the control group was injected with equivalent volume of NS. One month subsequent to the initial injections, mice in the aforementioned three groups were randomized into two subsets. One set was treated with the neurotoxin MPTP as second stimulus to establish the mouse model of PD with CIIM, while the other set received injections of NS. (Note: Repeated inflammatory stimuli (3xLPS or 4xLPS) yielded similar effects in mice.).

#### PD mouse model

Mice were weighed and then injected intraperitoneally with MPTP-HCL (M0896, Sigma, USA) at a dose of 25 mg/kg at the same time daily for 5 days. The control group was injected with NS.

### Cell culture and treatment

BV2 microglia cells and SH-SY5Y cells were cultured in DMEM medium containing 10% FBS. BV2 cells were seeded at a density of 5 × 10^4^ cells/well in 24-well plates for RNA or protein extraction. SH-SY5Y cells were seeded at a density of 1 × 10^4^ cells/well in 96-well plates for cell viability assay. 10 ng/mL or 100 ng/mL LPS were used as the first or second stimulus for BV2 cells.

### RNA extraction and quantitative real-time-PCR

Cell or tissue samples were lysed using TRNzol universal reagent (4992730, TIANGEN, China), and RNA extraction was performed according to the manufacturer's instructions. Concentration of the RNA was determined using a Microplate Spectrophotometer (Biotek, USA), and 1000 ng RNA was used to synthesize cDNA with a reverse transcription kit (CW2020, CWBIO, China). The target mRNA amount in the samples was detected using SYBR-Green (CW3008M, CWBIO, China) and quantitative real-time polymerase chain reaction (qPCR) on QuantStudio 5 Real-Time PCR Instrument (Applied Biosystems, USA). The relative level of mRNA was determined with β-actin as the normalizing control. The primers used for qPCR were listed in Additional file 1: Table S1.

### Protein extraction and western blot analysis

The cell or tissue samples were lysed using RIPA Buffer (P0013B, Thermo Fisher Scientific, USA) containing protease and phosphatase inhibitors (78425, Thermo Fisher Scientific, USA; B15001, B15002, Bimake, USA) and protein extraction was performed according to the manufacturer's instructions. Concentration of the protein was determined using the BCA protein assay kit (23225, Thermo Fisher Scientific, USA). Denatured protein diluted to an equal concentration was separated by SDS-PAGE. Subsequently, the protein was transferred to a PVDF membrane at a constant current of 300 mA. After blocked in 5% skim milk for 1 h, the membrane was incubated overnight with the primary antibody: rabbit anti-GFAP antibody (1:1000; 16825-1-AP, Proteintech, USA), mouse anti-HIF-1α antibody (1:200; SC-13515, Santa Cruz, USA), rabbit anti-IBA1 antibody (1:1000; ab178846, Abcam, USA), mouse anti-TH antibody (1:1000; 22941, Immunostar, USA), mouse anti-β-actin antibody (1:1000; SC-47778, Santa Cruz, USA). The next day, after washing, the membrane was incubated with the corresponding secondary antibody for 1 h, and the band of interest was detected using the Odyssey infrared imaging system (Li-Cor, USA). The relative level of protein was quantified using ImageJ software (NIH, Bethesda, MD).

### ELISA

Proteins were extracted from mouse striatum, and the concentrations were determined for ELISA analysis. The levels of IL-1β and IL-4 proteins were measured according to the manufacturer’s instructions (RK00006, RK00036, ABclonal, China).

### Immunofluorescence staining and immunohistochemical staining

Brain slices containing the target regions were washed in PBS. The Universal tissue fixative solution (BL539A, Biosharp, China) was used for post-fixation. After being blocked with 0.5% PBST containing 10% goat serum for 1 h, the slices were incubated overnight with the primary antibody: rat anti-CD16/32 antibody (1:200; 553142, BD Pharmingen, USA), rabbit anti-GFAP antibody (1:1000; 16825-1-AP, Proteintech, USA), mouse anti-HIF-1α antibody (1:50; NB100-105, Novus, USA), rabbit anti-IBA1 antibody (1:1000; ab178846, Abcam, USA), mouse anti-TH antibody (1:1000; 22941, Immunostar, USA). The next day, the corresponding fluorescent secondary antibody was incubated, and the images were captured using Confocal microscopy (Nikon, Japan). For immunohistochemical staining, the brain slices were pre-treated with 0.3% H_2_O_2_ after the post-fixation and incubated with biotinylated secondary antibodies (PK-4002, PK-6101, Vector Laboratories, USA). The signals were detected using the DAB Peroxidase (HRP) Substrate Kit (PK-6102, Vector Laboratories, USA) and captured using optical microscope (Olympus, Japan).

### Luminex assay of cytokines and chemokine

Tissue samples were lysed using ProcartaPlex Cell Lysis Buffer containing protease and phosphatase inhibitors. After determining the protein concentration using the method described above, the samples were diluted to 10 mg/ml with 1xPBS. The subsequent assays were performed according to the manufacturer’s instruction.

### Analysis of microglia morphology

The morphology of microglia was analyzed using ImageJ software (NIH, Bethesda, MD). Plugins in the software was used to convert the captured high-magnification images to binary format. After outlined and skeletonized, the soma area, endpoints and summed branch length of microglia were obtained [35].

### Behavioral tests

#### Pole test

Mice were placed with their heads upwards at the top of a rough and straight pole that was 80 cm long and equipped with a base. Time of turning and climbing down was recorded respectively to assess the motility of mice [36].

#### Rearing test

Mice are placed in a transparent beaker-shaped cylinder with a diameter of 8 cm and a height of 18 cm. During independent exploration, mice tended to rear up with forelimbs leaning upright against the wall. Spontaneous activity was monitored for 3 min, and the rearing times were counted [37].

#### Wire hanging test

Mice were placed with their forelimbs gripping the middle of a horizontal wire, which was 50 cm long and equipped with two platforms at both ends. The latency time on the wire was recorded for 3 min. Mice received one point for reaching a platform along the wire and lost one point for falling from the wire during test [38].

### Cell counting

For glial cell counting, 4 slices with a thickness of 30 μm were selected from each mouse. A frame of 700 μm × 700 μm was placed at the dorsal striatum or the substantia nigra compacta was outlined using ImageJ software. The numbers of cells within the frame were counted.

For stereological counting of TH-positive neurons in the substantia nigra, according to the sagittal position, 6 brain slices were selected at 10-slice intervals. Using the Stereo Investigator system (Micro Brightfield, USA), the total numbers of neurons were calculated by the supporting software in a double blinded manner.

### Statistical analysis

All data were analyzed with PRISM 9.0 (GraphPad Software, USA) and all values were presented as means ± SEM. Nonparametric test was used when the data did not fit normal distribution. For experiments involving only two groups, an unpaired t test was used for comparison. Depending on the number of independent variables, one-way or two-way ANOVA followed by LSD multiple comparison tests were used. *P* < 0.05 was considered to be statistically significant.

## Results

### Microglia have immune memory properties in vitro

**Fig. 1:**
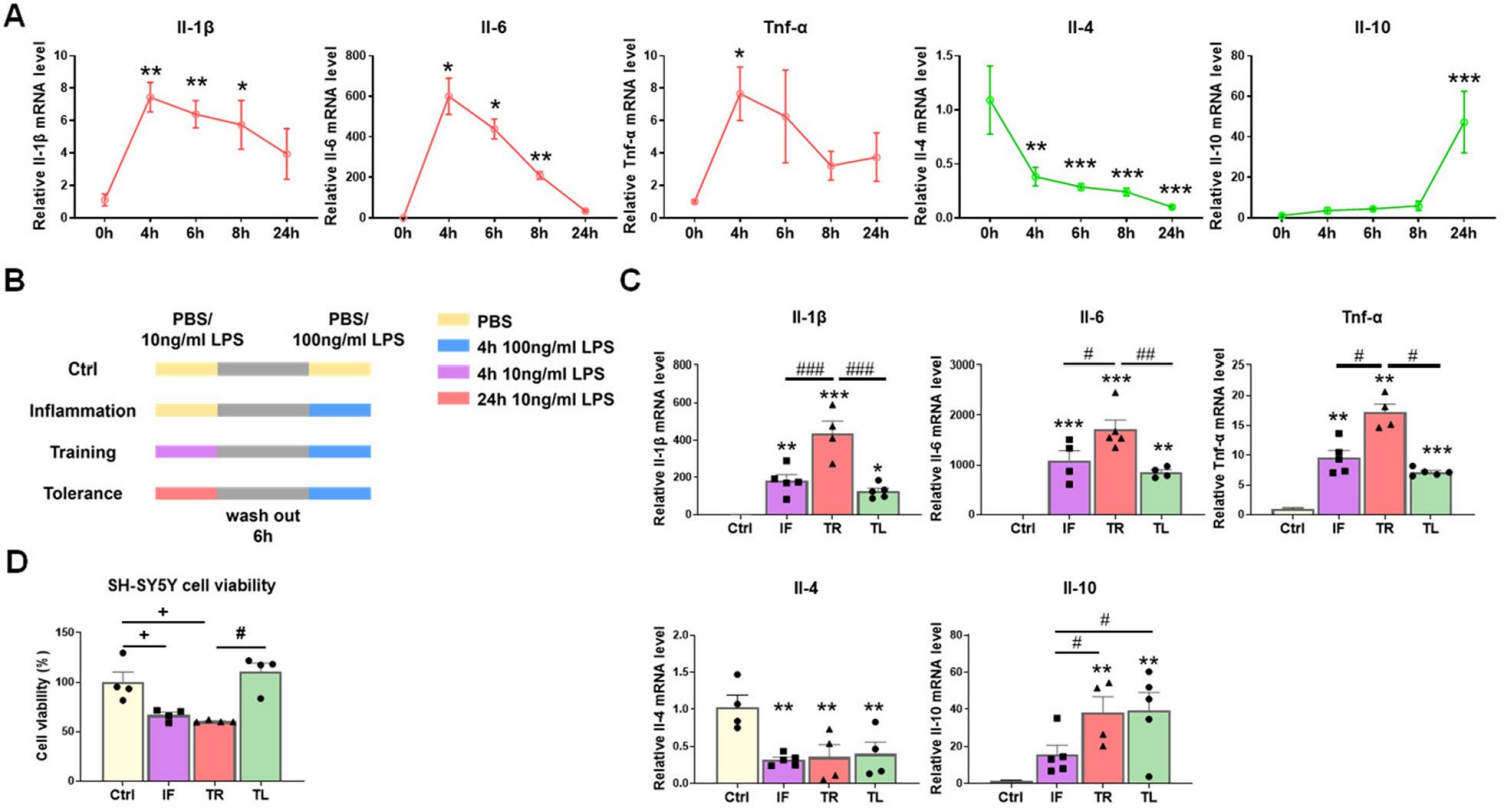
The Expression of inflammatory cytokines in LPS-treated BV2 microglial cells and the impact of their conditioned medium on the survival of SH-SY5Y cells. **A** Transcriptional changes of pro-inflammatory cytokines (*Il-1β, Il-6* and *Tnf-α*) and anti-inflammatory cytokines (*Il-4* and *Il-10*) in BV2 cells exposed to 10 ng/ml LPS for 0, 4, 6, 8 and 24 h. n = 3–4/group. **B** The experimental schedule diagram showing establishment of innate immune memory models in BV2 microglial cells. **C** Transcription levels of pro-inflammatory cytokines (*Il-1β, Il-6* and *Tnf-α*) and anti-inflammatory cytokines (*Il-4* and *Il-10*) in the BV2 microglial cells detected by RT-qPCR. n = 4–5/group. Ctrl, Control; IF, Inflammation; TR, Training; TL, Tolerance. **D** Cell viability of SH-SY5Y cells. The experimental procedure for LPS-pretreatment and the resting time interval were the same as **B**, conditioned medium (CM) was collected from BV2 cells after a 24 h-incubation with 100 ng/mL LPS. Cell viability was determined 48 h after incubated with CM collected from BV2 cells. n = 4/group. Differences were analyzed by one-way ANOVA followed by LSD multiple comparison tests. *p < 0.05, **p < 0.01, ***p < 0.001 vs control groups; ^#^*p* < 0.05, ^##^*p* < 0.01, ^###^*p* < 0.001. The difference shown by “+” was analyzed by unpaired t test. ^+^*p* < 0.05

Studies have reported that microglia acquired different inflammatory phenotypes upon repeated LPS stimulation in vitro [34, 39, 40]. To compare the different inflammatory responses following LPS preconditioning, we stimulated microglial BV2 cells with 10 ng/ml LPS for different durations and evaluated the transcriptional changes of inflammatory cytokines using RT-qPCR. The transcription levels of pro-inflammatory cytokines (*Il-1β, Il-6* and *Tnf-α*) peaked at 4 h and then decreased. In contrast, the levels of anti-inflammatory cytokines (*Il-4* and *Il-10*) exhibited different dynamic changes. After exposure to a low-dose of LPS, *Il-4* levels decreased, while *Il-10* levels showed a moderate increase from 0 to 8 h and a sharp increase at 24 h (Fig. 1A). Based on these findings, we designed an experimental schedule to observe whether the inflammatory states of BV2 cells following a low-dose LPS pretreatment for different time (first stimulus) influenced their responses to a subsequent high-dose LPS challenge (second stimulus). There were four experimental groups: Control (Ctrl), Inflammation (Inflammation, IF), innate immune training (Training, TR) and innate immune tolerance (Tolerance, TL) (Fig. 1B). We found that preconditioning BV2 cells to the peak of pro-inflammatory state exacerbated the subsequent high-dose LPS response even with a resting interval of 6 h, as indicated by dramatically higher expression of *Il-1β*, *Il-6* and *Tnf-α* compared to BV2 cells treated solely with LPS (IF group). However, BV2 cells preconditioned to an anti-inflammatory state somewhat maintained the anti-inflammatory properties, implied by increased transcriptional levels of Il-10 (Fig. 1C). These results suggested that BV2 microglial cells exhibited two forms of innate immune memory (IIM), of which the pro-inflammatory state induced-memory was termed innate immune training (TR) and the anti-inflammatory state induced-memory was termed innate immune tolerance (TL).

In addition, we assessed the potential impact of conditioned medium (CM) from BV2 cell models of IIM on the viability of dopaminergic SH-SY5Y cells. The findings revealed that the exposure to CM from IF group led to a decrease in cell survival. However, the viability of SH-SY5Y cells treated with CM from TL group was comparable to that of the control group. Interestingly, we observed higher cell viability after the treatment with TL CM compared to TR CM (Fig. 1D). These findings suggested that the CM from BV2 cell models of IIM exhibited diverse effects on the viability of SH-SY5Y cells; the IF group and TR group exerted detrimental effects and the TL group exhibited no harmful effect.

### Moderate LPS-induced inflammatory stimulation in the periphery regulates brain innate immune response in vivo

**Fig. 2:**
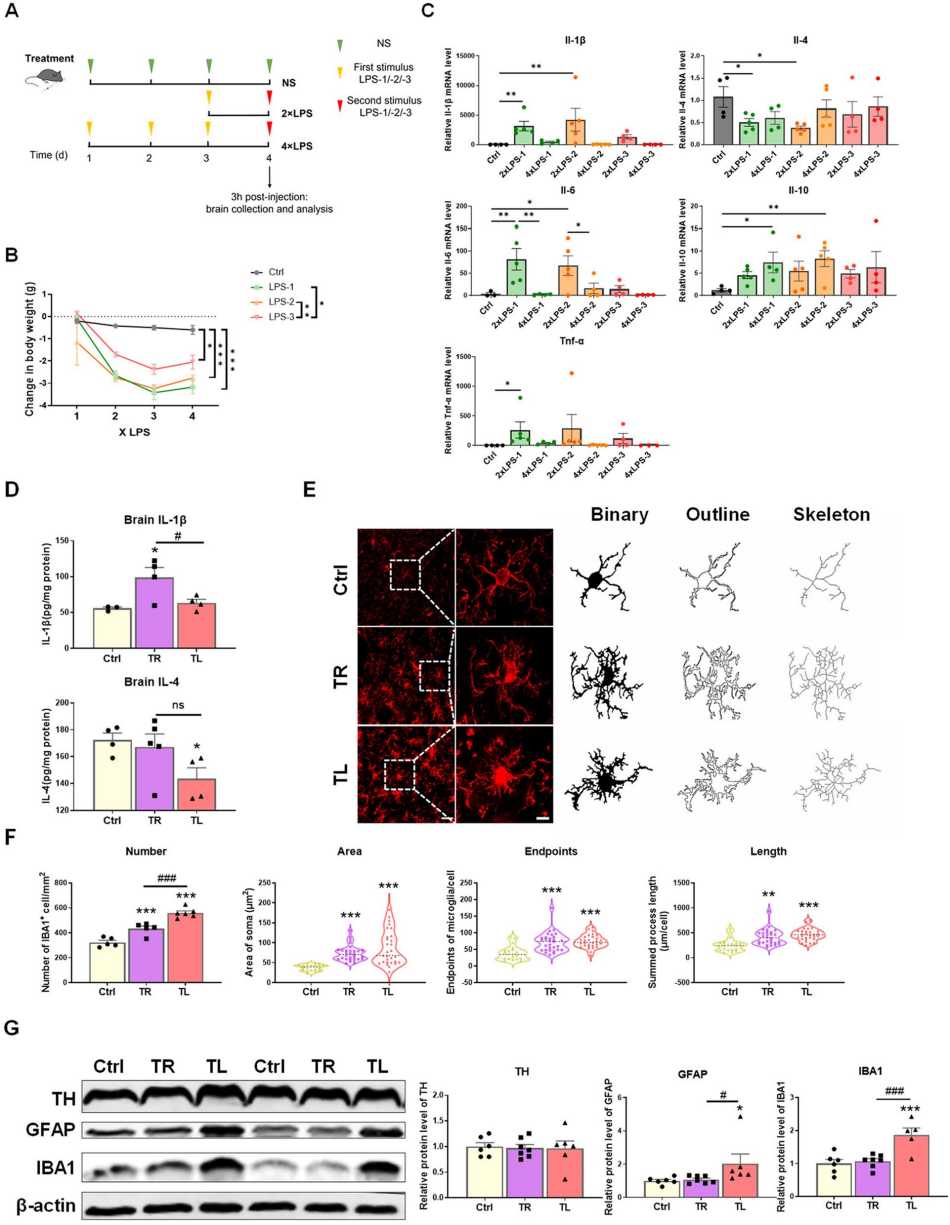
The innate immune responses in the brain of mice systemically challenged with different low doses of LPS. **A** The experimental schedule diagram showing establishment of innate immune memory models in mice. LPS-1: Lipopolysaccharides from *Salmonella enterica* serotype typhimurium; LPS-2: Lipopolysaccharides from *Escherichia coli* O111:B4; LPS-3: Lipopolysaccharides from *Escherichia coli* O26:B6. **B** The changes in body weight during different paradigms of LPS stimulation. n = 3–10 /group. Differences were analyzed by two-way ANOVA followed by LSD multiple comparison tests. *p < 0.05, **p < 0.01, ***p < 0.001. **C** Transcription of pro-inflammatory cytokines (*Il-1β, Il-6* and *Tnf-α*) and anti-inflammatory cytokines (*Il-4* and *Il-10*) in mouse striatum detected by RT-qPCR. n = 3–5/group. Differences were analyzed by one-way ANOVA followed by LSD multiple comparison tests. *p < 0.05, **p < 0.01. **D** Concentration of IL-1β and IL-4 proteins in the striatum of LPS-1 treated mice measured by ELISA. n = 3–5/group. Differences were analyzed by one-way ANOVA followed by LSD multiple comparison tests. *p < 0.05, vs control groups. #*p* < 0.05. **E** Representative images of IBA1 immunostaining in the striatum of mice injected with LPS-1. Scale bar, 25 µm. Microglial morphology was shown by zoomed representative images. Scale bar, 5 µm. **F** Quantification and morphology analysis of IBA1^+^ microglia in mouse striatum. n = 5–6 mice for each experimental group; N = 20–30 microglia for different groups. Differences were analyzed by one-way ANOVA followed by LSD multiple comparison tests(quantification) or Kruskal–Wallis test (morphology analysis). **p < 0.01, ***p < 0.001, vs control groups. ^###^*p* < 0.001. **G** Protein levels of TH, GFAP and IBA1 in mouse striatum detected by Western Blot. Quantification of relative TH, GFAP and IBA1 protein expression is shown in the right panel. n = 6–7/group. Differences were analyzed by one-way ANOVA followed by LSD multiple comparison tests. *p < 0.05, ***p < 0.001 vs control groups. ^#^*p* < 0.05, ^###^*p* < 0.001

Previous reports have demonstrated that microglia are main innate immune cells in brain and can retain innate immune memory (IIM) [41–43]. To explore the induction efficiency of innate immune memory of microglia in vivo, LPS extracted from three different kinds of Gram-negative bacteria was intraperitoneally injected daily to mice. LPS-1 (from *Salmonella enterica* serotype typhimurium), LPS-2 (from *Escherichia coli* O111:B4) and LPS-3 (from *Escherichia coli* O26:B6) were used in this study. First of all, we established mouse models of acute innate immune memory (AIIM) including Training (TR) and Tolerance (TL). The control group received 4 injections of normal saline (NS); The 2xLPS (1xLPS/1xLPS) group received LPS injections for two consecutive days, in which the first LPS injection serving as the first stimulus, and the second as the second stimulus; the 4xLPS group (3xLPS/1xLPS) received LPS injections for four consecutive days, in which the first three injections were regarded as the first stimulus and the fourth injection as the second stimulus (Fig. 2A). The changes in body weight during LPS injections were surveyed. The innate immune response of brain induced by the peripheral inflammatory stimuli was investigated by examining the transcriptional levels of pro- and anti-inflammatory cytokines in mouse striatum by RT–qPCR. We found that the immune response somewhat depended on the sources of LPS, despite the comparable trend in body weight losses (Fig. 2B, C). In the TR group, the levels of all the three pro-inflammatory cytokines (*Il-1β, Il-6* and *Tnf-α*) were significantly increased, whereas the expression of anti-inflammatory cytokine *Il-4* was decreased. The expression of *Il-10* transcript did not change compared to the control group. On the other hand, in the TL group, the transcriptional levels of pro-inflammatory cytokines remained low, and the expression of *Il-4* did not change. However, the expression of *Il-10* transcript was significantly elevated in this group (Fig. 2C). Among the three kinds of representative LPS, peripheral injection of LPS-1, but not LPS-2 and LPS-3, induced the most explicit AIIM in mouse brain. Moreover, the protein levels of IL-1β and IL-4 in mouse striatum were measured. We found that LPS-1 stimulation elicited a significant increase in IL-1β in TR group compared to the Control and TL groups. Conversely, there was a significant decrease in the level of IL-4 in TL group compared to the Control group, with no statistical difference observed between TR and TL groups (Fig. 2D). This means we confirmed the microglial IIM in vivo. By immunofluorescence staining of microglial marker IBA1 and cell counting, we found that the numbers of microglia were increased, and the area of soma, the numbers of endpoints, and the summed process length of microglia were significantly elevated as well, in the striatum of both TR and TL groups (Fig. 2E, F). Western blot results showed that the protein levels of IBA1 and GFAP in TL group were higher than those in the Control and TR groups, while TH protein levels remained unchanged (Fig. 2G). These results indicated that the patterns of brain IIM could be regulated by the systemic stimuli of different paradigms of LPS.

### HIF-1α plays a role in the regulation of brain innate immune memory

**Fig. 3:**
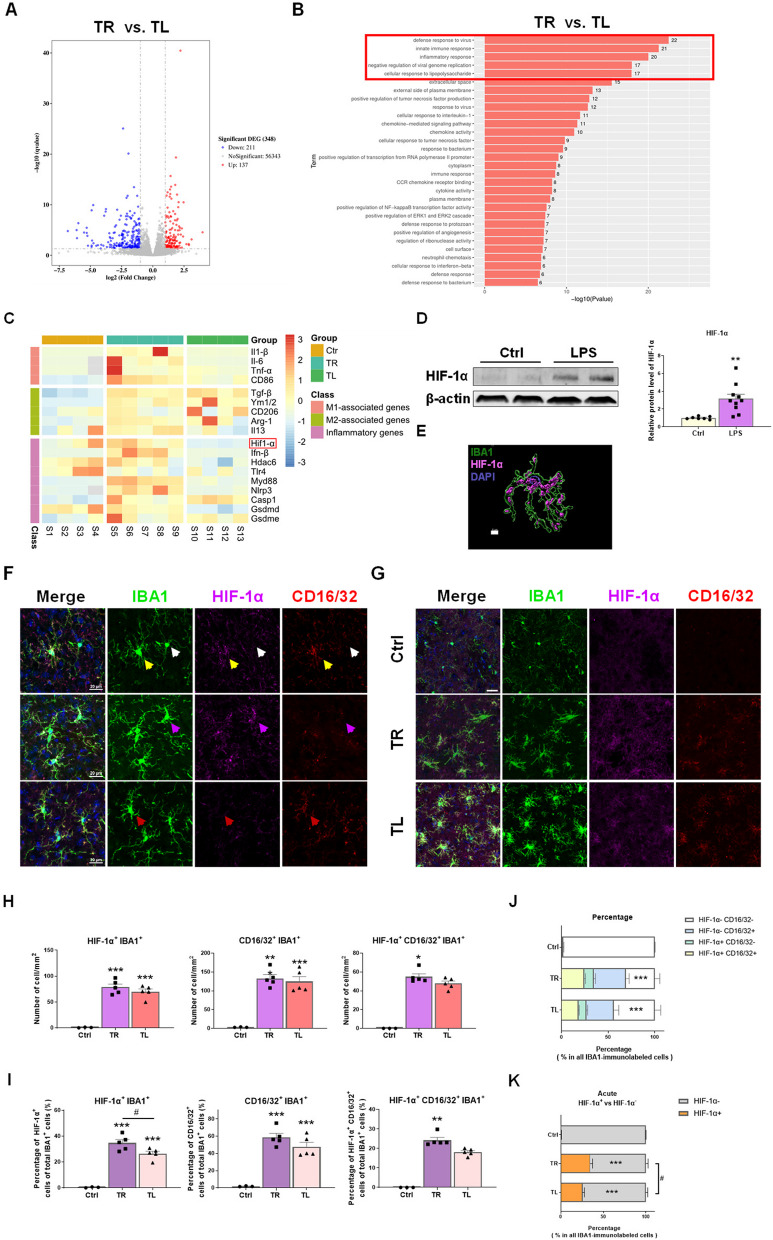

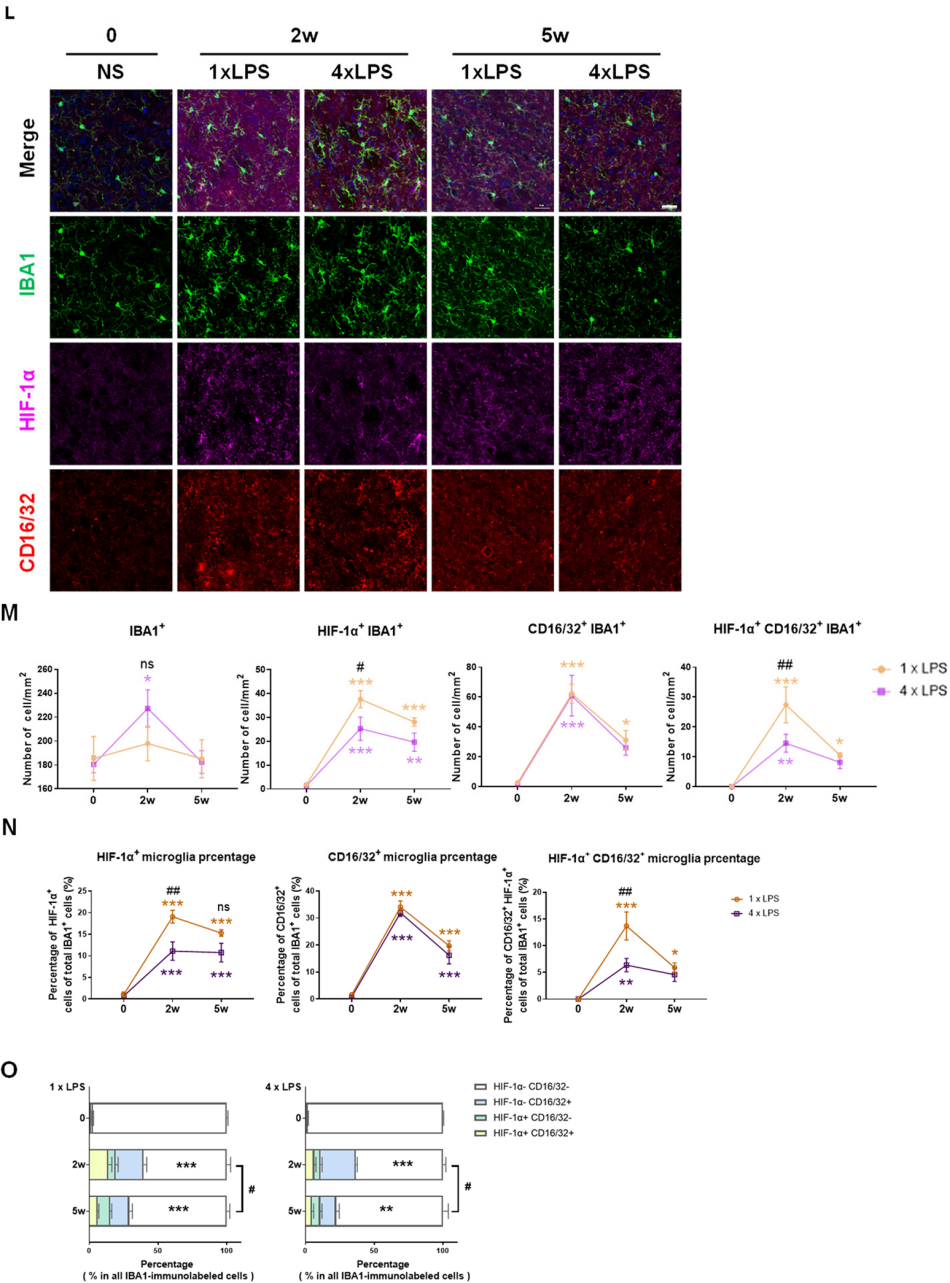
The expression of HIF-1α in microglia in innate immune memory models. **A**, **B** RNA-seq analysis of the striatum between mice treated with 2xLPS or 4xLPS. TR, Training; TL, tolerance. **A** The volcano plot of differentially expressed genes (DEGs). **B** The gene ontology (GO) enrichment analysis of DEGs. **C** The summary heatmap of RT-qPCR results based on M1/M2-associated genes and inflammatory genes in mouse striatum. n = 4–5/group. **D** Protein levels of HIF-1α in BV2 cells treated with LPS for 12 h, detected by Western Blot. n = 7–10/group. Differences were analyzed by unpaired t-test. **p < 0.01. **E** A representative 3D reconstruction image of activated microglia showing the colocalization of IBA1 (green) and HIF-1α (Magenta). Scale bar, 5 µm. **F** Co-localization of HIF-1α, CD16/32 and IBA1 in striatal microglia. Representative images from confocal microscopy revealed the colocalization of HIF-1α (Magenta) and CD16/32(red) in IBA1^+^ (green) microglia. White arrow: HIF-1α^−^ CD16/32^−^ cell; yellow arrow: HIF-1α^+^ CD16/32^+^ cell; magenta arrow: HIF-1α^+^ CD16/32^−^ cell; red arrow: HIF-1α^−^ CD16/32^+^ cell. Scale bar, 20 µm. **G** Representative images of IBA1**/**HIF-1α**/**CD16/32 triple immunostaining in mouse striatum. Scale bar, 50 µm. Small plots in the upper left quadrant showed the zoomed details. Scale bar, 20 µm. The experimental schedule was described as in Fig. 2A. (Ctrl, Control; TR, Training; TL, Tolerance). **H** Quantification of the numbers of HIF-1α^+^, CD16/32^+^ and HIF-1α^+^CD16/32^+^ cells among all the IBA1-immunolabeled cells. n = 3–5/group. Differences were analyzed by one-way ANOVA followed by LSD multiple comparison tests. *p < 0.05, **p < 0.01, ***p < 0.001, vs control groups. **I** The percentage of HIF-1α^+^, CD16/32^+^ and HIF-1α^+^ CD16/32^+^ cells within the total IBA1^+^ cells in mouse striatum. n = 3–5/group. Differences were analyzed by one-way ANOVA followed by LSD multiple comparison tests. **p < 0.01, ***p < 0.001, vs control groups. ^#^*p* < 0.05. **J** The relative proportion of sub-populations of IBA1-immunolabeled cells in the acute innate immune memory mouse model. The difference of HIF-1α^−^CD16/32^−^ microglia between groups was marked in the figure. n = 3–5/group. Differences were analyzed by one-way ANOVA followed by LSD multiple comparison tests. ***p < 0.001 vs control groups. **K** The relative proportion of IBA1-immunolabeled HIF-1α^+^ and HIF-1α^−^ cells in acute innate immune memory model. The difference in HIF-1α^−^ microglia between groups was marked in the figure. n = 4–5/group. Differences were analyzed by one-way ANOVA followed by LSD multiple comparison tests. *p < 0.05, **p < 0.01 vs control groups. #*p* < 0.05. **L** Representative images of IBA1**/**HIF-1α**/**CD16/32 triple immunostaining in the striatum of mice treated with NS, 1xLPS, or 4xLPS for two or five weeks. Scale bar, 50 µm. Small plots in the upper left quadrant showed the zoomed details. Scale bar, 20 µm. **M** Quantification of the numbers of HIF-1α^+^, CD16/32^+^ and HIF-1α^+^CD16/32^+^ cells in all IBA1-immunolabeled cells. n = 3–4/group. Differences were analyzed by two-way ANOVA followed by LSD multiple comparison tests. *p < 0.05, **p < 0.01, ***p < 0.001, vs control groups. ^#^*p* < 0.05, ^##^*p* < 0.01. **N** The percentages of HIF-1α^+^, CD16/32^+^ and HIF-1α^+^CD16/32^+^ in the total IBA1^+^ cells in mouse striatum. n = 3–4/group. Differences were analyzed by two-way ANOVA followed by LSD multiple comparison tests. *p < 0.05, **p < 0.01, ***p < 0.001, vs control groups. ^##^*p* < 0.01. **O** The relative proportions of HIF-1α^−^ CD16/32^−^ (white), HIF-1α^+^ CD16/32^+^ (yellow), HIF-1α^+^ CD16/32^−^ (green), and HIF-1α^−^ CD16/32^+^ (blue) in all IBA1-immunolabeled cells in acute innate immune memory mouse models. The difference of HIF-1α^−^ CD16/32^−^ microglia in 2w and 5w groups, compared to Control groups, was marked by asterisks (*) inside the figure, and difference of HIF-1α^−^ CD16/32^−^ microglia between 2 and 5w groups was indicated by pound sign (#). n = 3–4/group. Differences were analyzed by one-way ANOVA followed by LSD multiple comparison tests. **p < 0.01, ***p < 0.001, vs control groups. #*p* < 0.05

To assess whether the mouse striatum in TR and TL groups was characterized by a distinct gene expression profile, RNA-sequencing (RNA-seq) analysis of striatum from TR and TL mice collected at 3 h after LPS treatment was conducted. 137 genes were upregulated and 211 genes were downregulated in TL group compared to TR group (Fig. 3A). Gene ontology (GO) analysis showed the differentially expressed genes between the two groups were mainly concentrated in the cellular component, such as the cytoplasm and plasma membrane, and were involved in biological processes mainly related to defense response, innate immune response, inflammatory response, and cellular response to lipopolysaccharide (Fig. 3B, Additional file 1: Figure S1A). KEGG enrichment analysis showed that the enrichment pathways mainly focused on human diseases, environmental information processing and cellular processes including JAK-STAT signaling pathway (Additional file 1: Figure S1B). These results indicated that mouse brains acquired two types of IIM after the moderate LPS-induced inflammatory stimulation in the periphery. The findings from RNA-seq were further confirmed by RT-qPCR assays. On the whole, the transcription of M1-associated genes, including *Il-1β, Tnf-α* and *CD86*, was increased in TR group compared to Control group. However, the transcription of M2-associated genes (*Tgf-β, Ym1/2*) was increased both in TR group and TL groups*,* whereas *CD206, Arg-1* and *Il-13* transcripts were comparable among Control, TR and TL groups. The differences in the transcription of inflammatory genes including *Hif-1α, Ifn-β, Hdac6, Myd88 and Nlrp3* between TR and TL groups further demonstrated that the signal pathways involved were distinct (Fig. 3C; Additional file 1: Figure S2). Several studies have reported that HIF-1α played a crucial role in immune inflammatory response [25, 28, 44]. By analyzing the protein level of HIF-1α in LPS-treated BV2 cells, we found that HIF-1α responded to inflammatory stimulation in microglia (Fig. 3D). Subsequent double-immunofluorescence staining for HIF-1α and IBA1 further confirmed that HIF-1α was expressed in activated microglia in mice (Fig. 3E). In view of the above results, CD16/32 was chosen as an indicator of pro-inflammatory phenotypic marker to explore the involvement of HIF-1α in microglial activation state. As depicted in Fig. 3F, all IBA1-immunopositive cells were classified into four clusters: HIF-1α^−^ CD16/32^−^ (white arrow, expressing neither HIF-1α nor CD16/32), HIF-1α^+^ CD16/32^+^ (yellow arrow, expressing both HIF-1α and CD16/32), HIF-1α^+^ CD16/32^−^ (magenta arrow, expressing HIF-1α but not CD16/32), HIF-1α^−^ CD16/32^+^ (red arrow, expressing CD16/32 but not HIF-1α).

Firstly, the changes of microglial phenotype in AIIM mouse models, established according to the method in Fig. 2A, were examined. Triple immunofluorescence staining of IBA1, HIF-1α and CD16/32 in mouse striatum, followed by cell counting, was performed (Fig. 3G). The results showed that the numbers of each cell type, including HIF-1α^+^ IBA1^+^ cells, CD16/32^+^ IBA1^+^ cells and HIF-1α^+^ CD16/32^+^ cells, were markedly increased in both the TR and TL groups, although no difference was observed between the two groups (Fig. 3H). Notably, the percentage of HIF-1α^+^ microglia in TL group was decreased compared with TR group (Fig. 3I). The different proportion of four types of microglia (HIF-1α^−^ CD16/32^−^ microglia, HIF-1α^+^ CD16/32^+^ microglia, HIF-1α^+^ CD16/32^−^ microglia and HIF-1α^−^ CD16/32^+^ microglia) showed the change of microglial molecular signatures in LPS-injected mice (Fig. 3J). Given the sharp increase in the number of microglia in TL group compared to TR group (Fig. 2E and data not shown), we further investigated the relative percentage of IBA1-immunolabeled HIF-1α^+^ and HIF-1α^−^ cells in AIIM model and found a significant differences in the proportion of HIF-1α^−^ microglia between these groups (Fig. 3K). The results indicated that HIF-1α^−^ microglia constituted the majority of the increased cell population in TL group.

To further investigate the persistence of the phenotype in microglia, we conducted triple immunofluorescence staining for IBA1, HIF-1α and CD16/32 in the striatum of mice received training- and tolerance-inducing stimuli for two or five weeks (Fig. 3L). The results revealed that the numbers of HIF-1α^+^, CD16/32^+^ and HIF-1α^+^ CD16/32^+^ microglia were higher in the training-induced group (1xLPS) compared than those in the Control group over time. Similarly, in the tolerance-induced group (4xLPS), numbers of these cell types were higher than those of the Control group at 2 weeks but decreased over time to reach similar levels as the Control group. Notably, 2 weeks post-LPS injection, there was a significant difference in the numbers of HIF-1α^+^ and HIF-1α^+^ CD16/32^+^ cells between the 1xLPS and 4xLPS groups (Fig. 3M). Furthermore, the dynamic trends in the proportions of HIF-1α^+^, CD16/32^+^ and HIF-1α^+^ CD16/32^+^ microglia were consistent with the observed quantity trends (Fig. 3N). The differences in the proportion of HIF-1α^−^ CD16/32^−^ microglia between different groups were remarkable (Fig. 3O). The relative proportions of each type of cell revealed that the phenotypic transformation of microglia persisted for weeks, even as the density of microglia returned to control levels.

### Innate immune memory can last for one month and influence MPTP-induced immune response

**Fig. 4:**
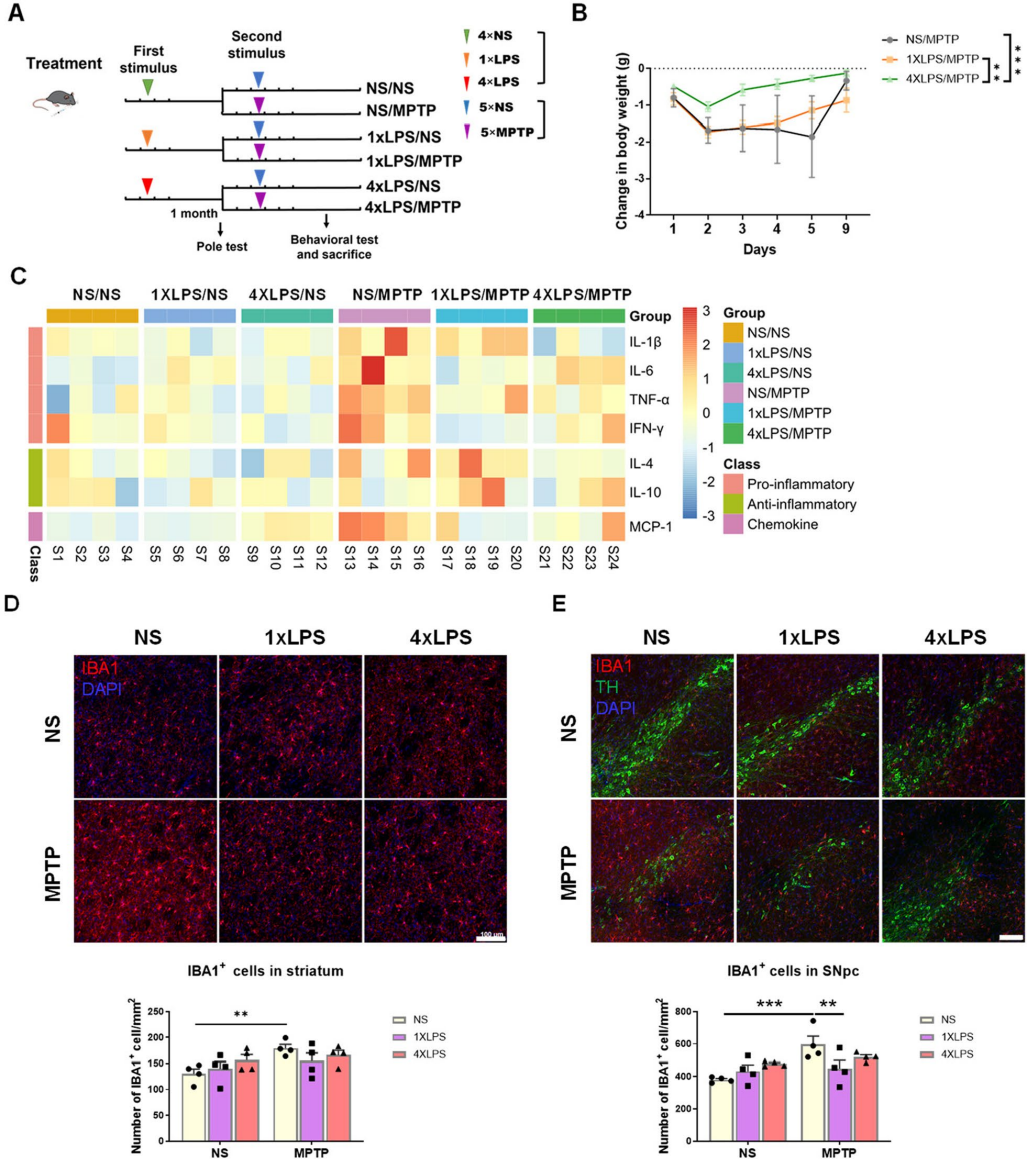
MPTP-induced immune responses in mouse brain can be attenuated to a certain degree one month after intraperitoneal injections of low-dose(s) of LPS. **A** The experimental schedule diagram illustrating the establishment of MPTP-induced mouse model of Parkinson’s disease following the induction of IIM. **B** The changes in body weight during and after MPTP injections. n = 6–7 /group. Differences were analyzed by two-way ANOVA followed by LSD multiple comparison tests. **p < 0.01, ***p < 0.001. **C** The summary heatmap of protein levels based on Luminex assay of pro-inflammatory cytokines (IL-1β, IL-6, TNF-α and IFN-γ), anti-inflammatory cytokines (IL-4 and IL-10) and chemokine (MCP-1) in the striatum of mice at 3 days post-saline or MPTP treatment. n = 4/group. **D**, **E** Assessments of microglial activation in the nigrostriatal pathway. Immunofluorescence staining of IBA1 (red) in the striatum (**D**) and immunofluorescence double staining of TH (green) and IBA1 (green) in the substantia nigra (**E**) at 7 days post-saline or MPTP administration. Scale bar, 100 μm. Quantification of IBA1^+^ cells in the dorsal striatum and the SNpc is shown in the bottom panel. n = 4/group. Differences were analyzed by two-way ANOVA followed by LSD multiple comparison tests. **p < 0.01, ***p < 0.001

The state of microglia in the brain may influence the pathology of CNS disorders [45, 46]. Studies have shown that the pathological process of AD can be regulated by stimuli applied months before [12]. To further investigate whether the sustained microglial IIM had an impact on PD-like pathology, we established the MPTP-induced mouse model of PD based on the IIM models. As indicated in the experimental schedule diagram, there was a one-month interval between the training- and tolerance-inducing stimuli (1x or 4xLPS, first stimulus) and the MPTP administration (second stimulus) (Fig. 4A). Because the interval between the first stimulus and the second stimulus in this model was one month, we called it chronic innate immune memory model (CIIM). Among them, mice in 1xLPS/MPTP group were PD mice with innate immune training (short for TR PD mice) and mice in 4xLPS/MPTP group were PD mice with innate immune tolerance (short for TL PD mice). The injection of LPS caused a momentary weight loss followed by a recovery before MPTP administration (Additional file 1: Figure S3A). In addition, the Pole test results showed that low dose(s) of LPS did not cause motor dysfunction (Additional file 1: Figure S3B). After MPTP injection, mice in 4xLPS/MPTP group exhibited the smallest weight changes compared with mice in 1xLPS/MPTP and NS/MPTP groups (Fig. 4B). MPTP administration alone triggered an inflammatory response [47–50]. Luminex assay was conducted to determine the protein levels of cytokines and chemokine in the striatum of mice at 3 days after MPTP treatment. Compared to the NS/NS group, the levels of IL-1β, IL-6, TNF-α and MCP-1 in the striatum of NS/MPTP group were significantly increased, but the levels of IFN-γ and IL-4 were not significantly different from those of NS/NS group. Compared with 1xLPS/NS group, the expression of IL-1β and IL-4 proteins in the striatum of 1xLPS/MPTP group increased, while the levels of IL-6, IFN-γ and MCP-1 proteins decreased. Only the level of IL-6 protein in the striatum of 4xLPS/MPTP group was higher than that of 4xLPS/NS group. The levels of IL-1β, TNF-α, IFN-γ, IL-4 and MCP-1 proteins in 4xLPS/MPTP group were similar to those in 4xLPS/NS group, while the expression of IL-1β, TNF-α and MCP-1 proteins was significantly lower than that of NS/MPTP group. In addition, there was no significant difference in IL-10 protein expression among all experimental groups (Fig. 4C, Additional file 1: Figure S3C). On the whole, the results suggested that the expression levels of inflammatory factors were altered dependent on the first stimulus one month ago, and compared to the NS/MPTP and 1xLPS/MPTP group, mice in 4xLPS/MPTP group exhibited the smallest changes in cytokine protein levels in the striatum. Furthermore, immunostaining for IBA1 in the striatum and SNpc of mice showed that MPTP-induced increase in the number of IBA1^+^ cells was reversed due to the IIM provoked by the first stimulus (Fig. 4D, E).

### Altered MPTP toxicity in the nigrostriatal pathway of mice acquired innate immune memory

**Fig. 5:**
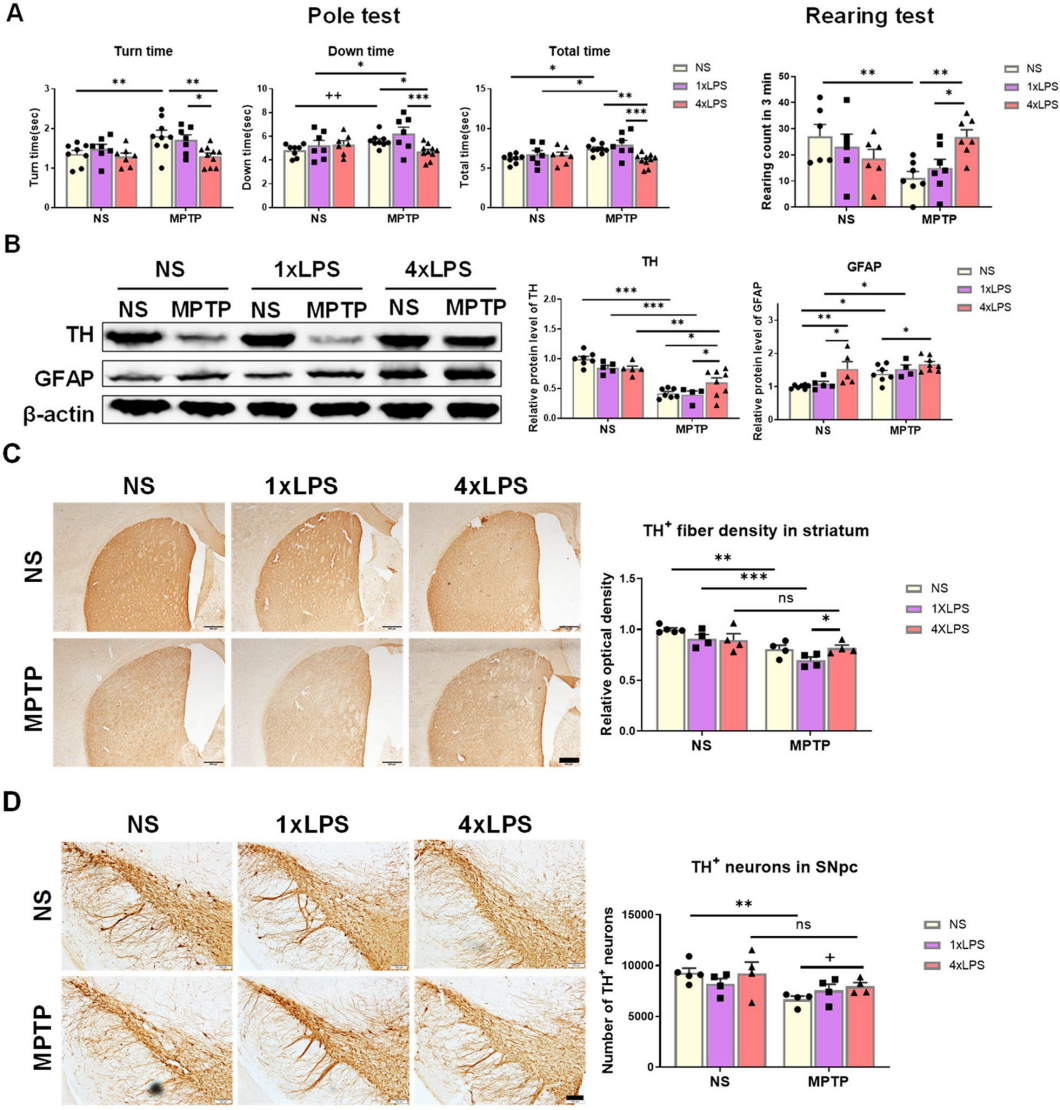
The pre-stimuli of 1xLPS or 4xLPS change MPTP-induced motor impairments and dopaminergic damage to the nigrostriatal axis in mice. **A** The results of behavioral tests in mice 2 days post-MPTP administration. Time to turn around, climb down and the total time in the Pole test, and the rearing count over a 3-min period in the Rearing test were shown. n = 6–10/group. The difference shown by “++” was analyzed by unpaired t test. ^++^*p* < 0.01. **B** The protein levels of TH and GFAP in mouse striatum detected by Western Blot. n = 4–8 /group. **C** Assessments of the optical density of TH^+^ nerve fibers in the striatum of mice by immunohistochemistry staining. Scale bar, 200 μm. n = 4/group. **D** Assessments of TH^+^ neurons in the SNpc of mice by immunohistochemistry staining and stereological counting. Scale bar, 100 μm. n = 4/group. Differences were analyzed by two-way ANOVA followed by LSD multiple comparison tests. *p < 0.05, **p < 0.01, ***p < 0.001. The difference shown by “+” was analyzed by unpaired t test. ^+^*p* < 0.05. ns, no significant difference

Given that mice exposed to MPTP exhibit movement disorders and damage to the nigrostriatal pathway [36, 49, 51], we investigated whether IMM had any effects on PD-like behaviors and pathology. The Pole test results showed that mice treated with MPTP alone took longer to turn around, climb down, and complete the entire process. Mice in 1xLPS/MPTP group also displayed some motor impairment, as evidenced by the increased time to climb down and total time spent. However, mice in 4xLPS/MPTP group showed no motor impairment compared to NS group, and they performed significantly faster than those in NS/MPTP and 1xLPS/MPTP groups. Similarly, the results of the Rearing test showed that there was a significant decrease in the number of rearing counts in NS/MPTP group compared to NS group. However, such a decline was reversed in 4xLPS/MPTP group (Fig. 5A). Western blotting and immunohistochemistry assays were conducted to assess the damages of dopaminergic system in nigrostriatal pathway. Treatment with MPTP in mice caused a decreased in striatal TH protein levels in all the groups regardless of the pre-stimulations. However, TH levels in both NS/MPTP and 1xLPS/MPTP groups were significantly reduced compared to 4xLPS/MPTP group (Fig. 5B). By assessing the optical density of TH^+^ nerve fibers in the striatum and TH^+^ neurons in the SNpc, we observed that MPTP treatment led to a depletion of striatal TH^+^ nerve fibers in mice of NS/MPTP and 1xLPS/MPTP groups. Additionally, MPTP treatment resulted in a reduction of TH^+^ neurons only in mice of NS/MPTP group. However, pre-stimulation with 4xLPS elicited resistance to MPTP-induced dopaminergic damage both in the striatum and SNpc, as indicated by the elevated TH^+^ nerve fibers and TH^+^ neurons in 4xLPS/MPTP group compared to 1xLPS/MPTP and NS/MPTP group, respectively. Moreover, mice in 1xLPS/MPTP group also showed no dramatical loss of TH^+^ neurons in the SNpc (Fig. 5C, D). These results revealed that 4xLPS alleviated the dopaminergic neuronal loss, while the effect of 1xLPS was limited. Since there exists bi-directional communication between microglia and astrocytes, the astrocyte reactivity was assessed by western blot and immunohistochemical staining. The levels of striatal GFAP proteins and the number of GFAP^+^ cells in the NS/MPTP and 1xLPS/MPTP groups were increase. Striatal level of GFAP proteins in the 4xLPS group did not restore to the normal level by the time of the second stimulus (MPTP) administration and was significantly increased compared to NS and 1xLPS groups. However, MPTP treatment did not further upregulate the expression of GFAP proteins in 4xLPS/MPTP group (Fig. 5B). Interestingly, the number of GFAP^+^ cells in the striatum was significantly higher in 4xLPS/MPTP group compared to 4xLPS/NS group, and there were no differences observed among the three groups that received MPTP injections (Additional file 1: Figure S4). These results indicated that the innate immune tolerance memory lasted for at least one month and led to a certain resistance against dopaminergic degeneration in PD mouse model. However, the modified microglia in TR group did not induce aggravated PD-like pathology in mice.

### HIF-1α plays a role in the MPTP-induced inflammation in chronic innate immune memory mouse models

**Fig. 6:**
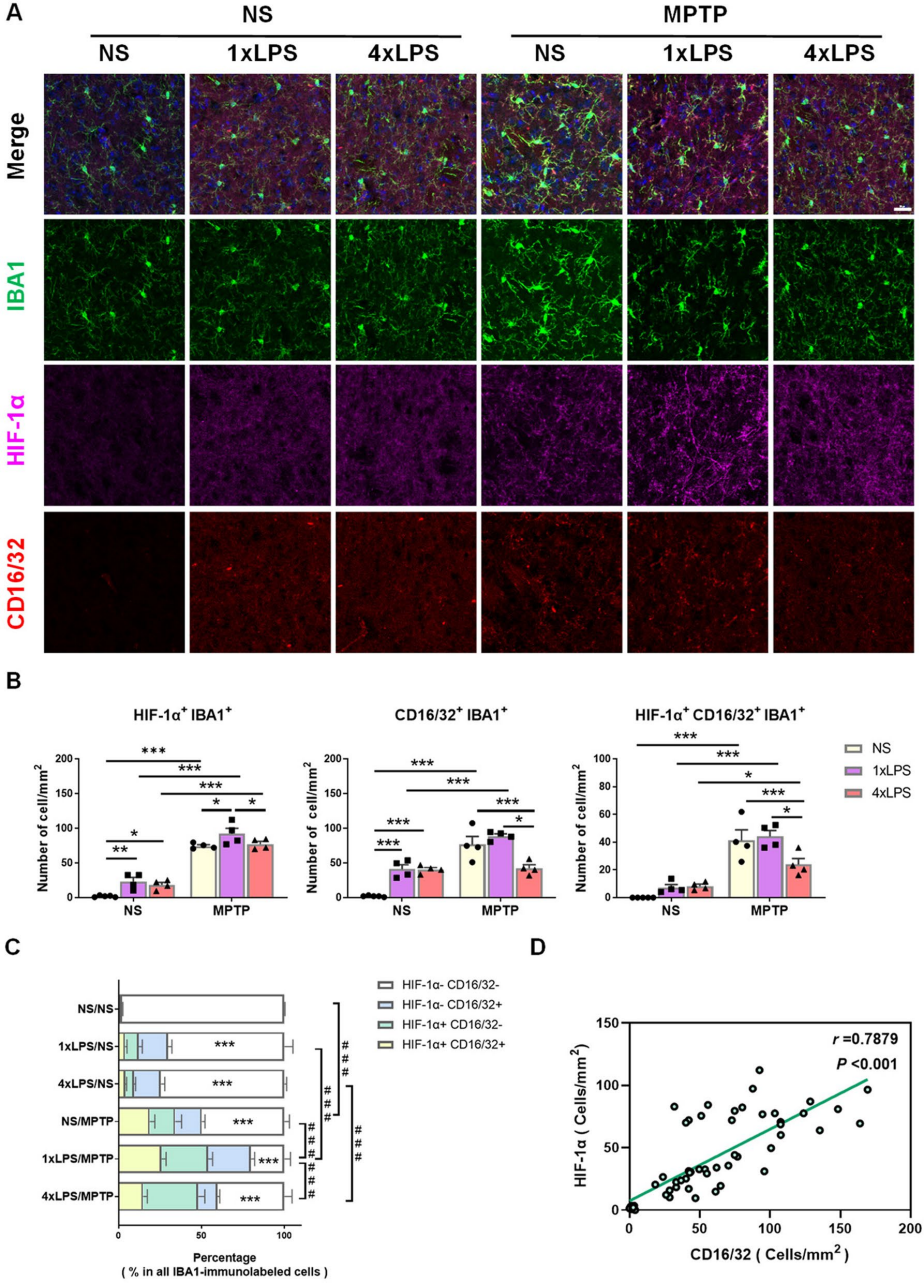
The expression of HIF-1α in striatal microglia of chronic innate immune memory model mice treated with NS or MPTP. **A** Representative images of IBA1**/**HIF-1α**/**CD16/32 triple immunostaining in the striatum of MPTP-induced PD mice with innate immune memory. Scale bar, 50 µm. Small plots in the upper left quadrant showed the zoomed details. Scale bar, 20 µm. The experimental schedule was described as in Fig. 3A, B. Quantification of the number of HIF-1α^+^, CD16/32^+^ and HIF-1α^+^ CD16/32^+^ cells in all IBA1-immunolabeled cells from mouse striatum, 7 days post-MPTP administration. n = 4/group. Differences were analyzed by two-way ANOVA followed by LSD multiple comparison tests. *p < 0.05, **p < 0.01, ***p < 0.001. **C** The relative proportion of IBA1-immunolabeled cells in MPTP-induced PD mice based on innate immune memory models. The changes in the proportion of HIF-1α^−^ CD16/32^−^ microglia in the experimental groups compared to Control groups was marked by asterisks (*) within the figure, and differences between these groups was indicated by pound sign (#). n = 4/group. Differences were analyzed by one-way ANOVA followed by LSD multiple comparison tests. *p < 0.05, **p < 0.01, ***p < 0.001 vs control groups. ^###^*p* < 0.001. **D** Pearson’s correlation analysis showing density of HIF-1α^+^ and CD16/32^+^ cells in all of the IBA1-immunolabeled microglia. Pearson's correlation coefficient (*r*) and *p*-values are shown above

According to the above experimental results, the existence of innate immune tolerance memory alleviated the MPTP-induced damage in nigrostriatal pathway. In view of the regulating effect of HIF-1α on IIM, we investigated the activation state of microglia in PD mouse models with IIM. The experimental schedule followed what was described in Fig. 4A. Results of triple immunofluorescent staining and the subsequent quantification analysis revealed an increased density of HIF-1α^+^ microglia and CD16/32^+^ microglia in 1xLPS or 4xLPS groups, compared to NS group, one month after the pre-stimulation. MPTP administration increased the number of HIF-1α^+^ microglia and HIF-1α^+^ CD16/32^+^ microglia in all groups, and increased the number of CD16/32^+^ microglia in NS/MPTP (PD) and 1xLPS/MPTP (TR PD) groups, but not in the 4xLPS/MPTP (TL PD) group. After the second stimulus of MPTP, mice in TL PD group displayed reduced density of CD16/32^+^ microglia and HIF-1α^+^ CD16/32^+^ microglia compared to both the PD and TR PD groups. At the same time, the TR PD mice exhibited an increased number of HIF-1α^+^ microglia compared to PD and TL PD mice (Fig. 6A, B). After analyzing the relative proportion of microglia subpopulations in MPTP-induced PD mice based on IIM models, we found there were significant variations. The differences in the proportion of HIF-1α^−^ CD16/32^−^ microglia between these groups were remarkable. Compared to NS group, the proportions of HIF-1α^−^ CD16/32^−^ microglia were lower in all the other groups. Administration of MPTP resulted in a decrease in the proportion of HIF-1α^−^ CD16/32^−^ microglia compared to their respective control groups (NS, 1xLPS or 4xLPS). Among the three groups intoxicated with MPTP, 1xLPS/MPTP group exhibited the lowest proportion of HIF-1α^−^ CD16/32^−^ microglia compared to the other two groups (Fig. 6C). Considering all of the quantitative results from the HIF-1α and CD16/32 staining, we conducted a correlation analysis to assess the density of HIF-1α^+^ and CD16/32^+^ cells among all the IBA1-immunolabeled microglia. Correlation diagram (scatter plot) revealed a significant positive correlation with an *r* value of 0.7879, *p* < 0.001 (Fig. 6D). Altogether, these results suggested the expression of HIF-1α in microglia had correlation with CD16/32 and could serve as a biomarker for assessing microglia phenotype.

### Specific knockout of Hif-1α in microglia hinders the formation of innate immune memory

**Fig. 7:**
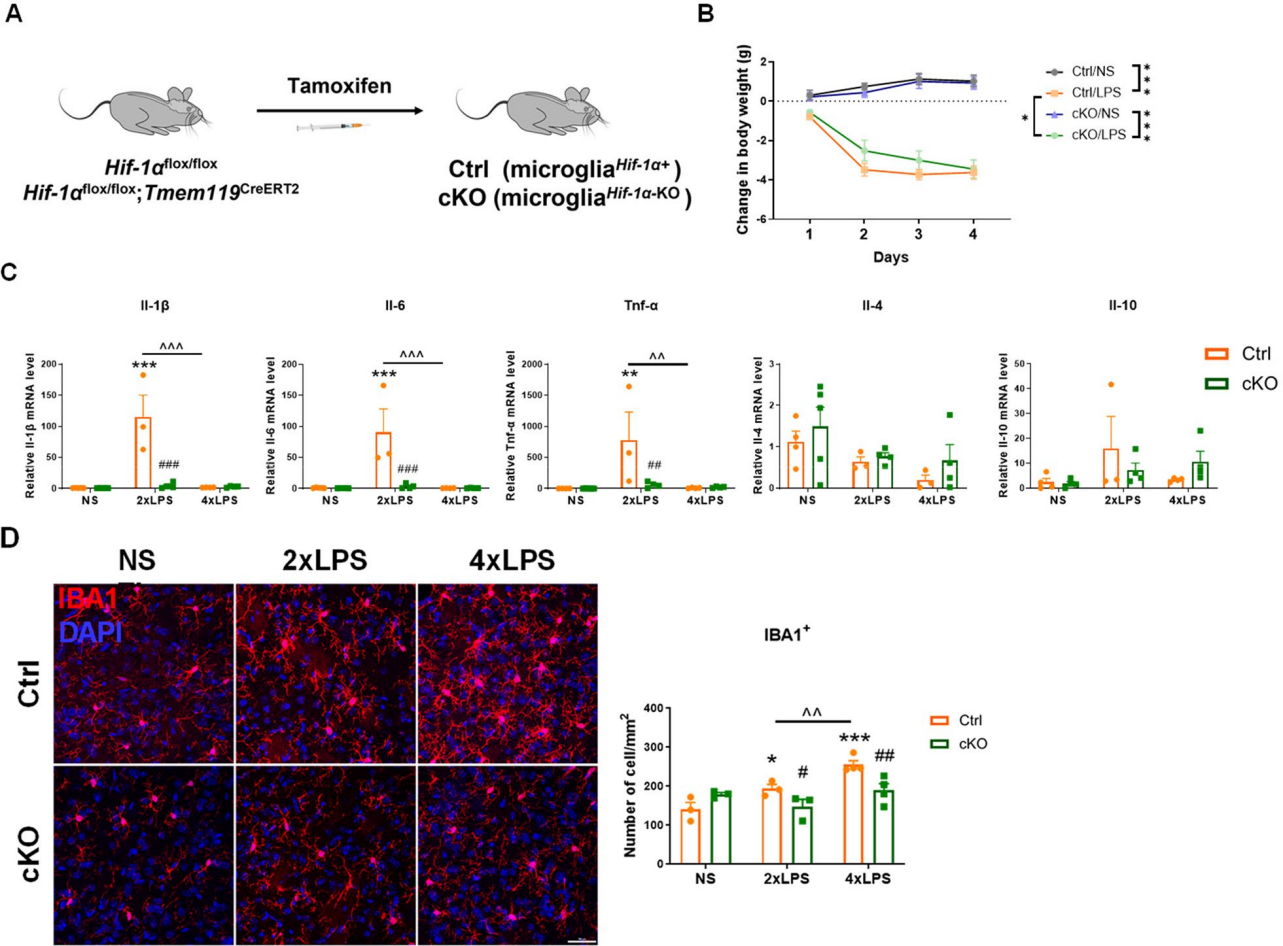
Acute innate immune memory models cannot be established in microglial *Hif-1α* conditional knockout mice. **A** Experimental strategy for tamoxifen-induced deletion of *Hif-1α* specifically in microglia. **B** The changes of body weight during LPS injections. n = 5–8/group. Differences were analyzed by two-way ANOVA followed by LSD multiple comparison tests. *p < 0.05, ***p < 0.001. The experimental schedule was the same as Fig. 2A. (**Ctrl**: Cre-negative control littermates; **cKO**: *Hif-1α* cKO). **C** Transcription of pro-inflammatory cytokines (*Il-1β*, *Il-6* and *Tnf-α*) and anti-inflammatory cytokines (*Il-4* and *Il-10*) in the striatum of Ctrl and cKO mice detected by RT-qPCR. n = 3–5 /group. Differences were analyzed by two-way ANOVA followed by LSD multiple comparison tests. **p < 0.01, ***p < 0.001 vs normal saline (NS) control. ^##^*p* < 0.05, ^###^*p* < 0.001 vs Ctrl mice. ^^*p* < 0.01, ^^^*p* < 0.001. **D** Representative images of IBA1 (red) immunostaining in the striatum of mice 3 h after NS, 2xLPS or 4xLPS injection. Scale bar, 100 µm. Quantity analysis of IBA1^+^ cells in the dorsal striatum is shown in the right panel. n = 3–4/group. Differences were analyzed by two-way ANOVA followed by LSD multiple comparison tests. *p < 0.05, ***p < 0.001 vs normal saline (NS) control. #*p* < 0.05, ##*p* < 0.01 vs Cre-negative control. ^^*p* < 0.01

To further explore the role of HIF-1α in innate immune memory regulation, we employed a tamoxifen-inducible *Hif-1α* conditional knockout (cKO) mouse model whereby *Hif-1α* was selectively deleted in TMEM119^+^ microglia. Mice were treated with tamoxifen for 5 consecutive days, and the subsequent experiments were conducted 2 weeks later. We performed PCR and immunofluorescence staining to examine the absence of microglial HIF-1α in cKO mice. Notably, HIF-1α was rarely expressed under physiological conditions (Fig. 7A, Additional file 1: Figure S5A-C). Our results confirmed the high efficiency of *Hif-1α* gene deletion in microglia.

To investigate whether lack of HIF-1α in microglia had any effects on IIM, we established AIIM models in Cre-negative control (Ctrl) and *Hif-1α* cKO mice. The experimental schedule diagram was the same as Fig. 2A. We found that cKO mice exhibited less weight loss compared to controls during LPS injections (Fig. 7B). When compared to Ctrl mice, transcriptional levels of pro-inflammatory cytokines, including *Il-1β*, *Il-6* and *Tnf-α*, in the striatum of cKO mice treated with 2xLPS were significantly inhibited. On the other hand, the transcription of these cytokines in the striatum of both Ctrl and cKO mice following the stimuli of 4xLPS did not change, in comparison to the NS group, and there was no significant difference between the two 4xLPS-treated groups (Fig. 7C). Notably, the expression of inflammatory cytokines in NS-treated Ctrl and cKO mice showed no difference. By analyzing the above results, we observed that the deficiency of HIF-1α in microglia hindered the formation of TR, as evidenced by the impaired response of mice to inflammatory stimuli. Since the formation of TL depended on the microglial immune response capability, we speculated that the attenuated immune response in Ctrl mice after the stimulation of 4xLPS was a consequence of TL formation. Nevertheless, the relative insensitive state of microglia caused by the deficiency of HIF-1α may predominantly account for the diminished response following 4xLPS stimulation. We also conducted immunofluorescence staining of IBA1 to investigate the quantity of microglia. The results showed that microglial HIF-1α deficiency inhibited LPS-induced increase in microglia density in all the two groups (Fig. 7D). This suggesting that microglial HIF-1α mediated innate immune response and shaped IIM in the brain.

### Microglial HIF-1α regulates cell phenotypic polarization and inflammatory response in the MPTP-induced mouse model of Parkinson’s disease

**Fig. 8:**
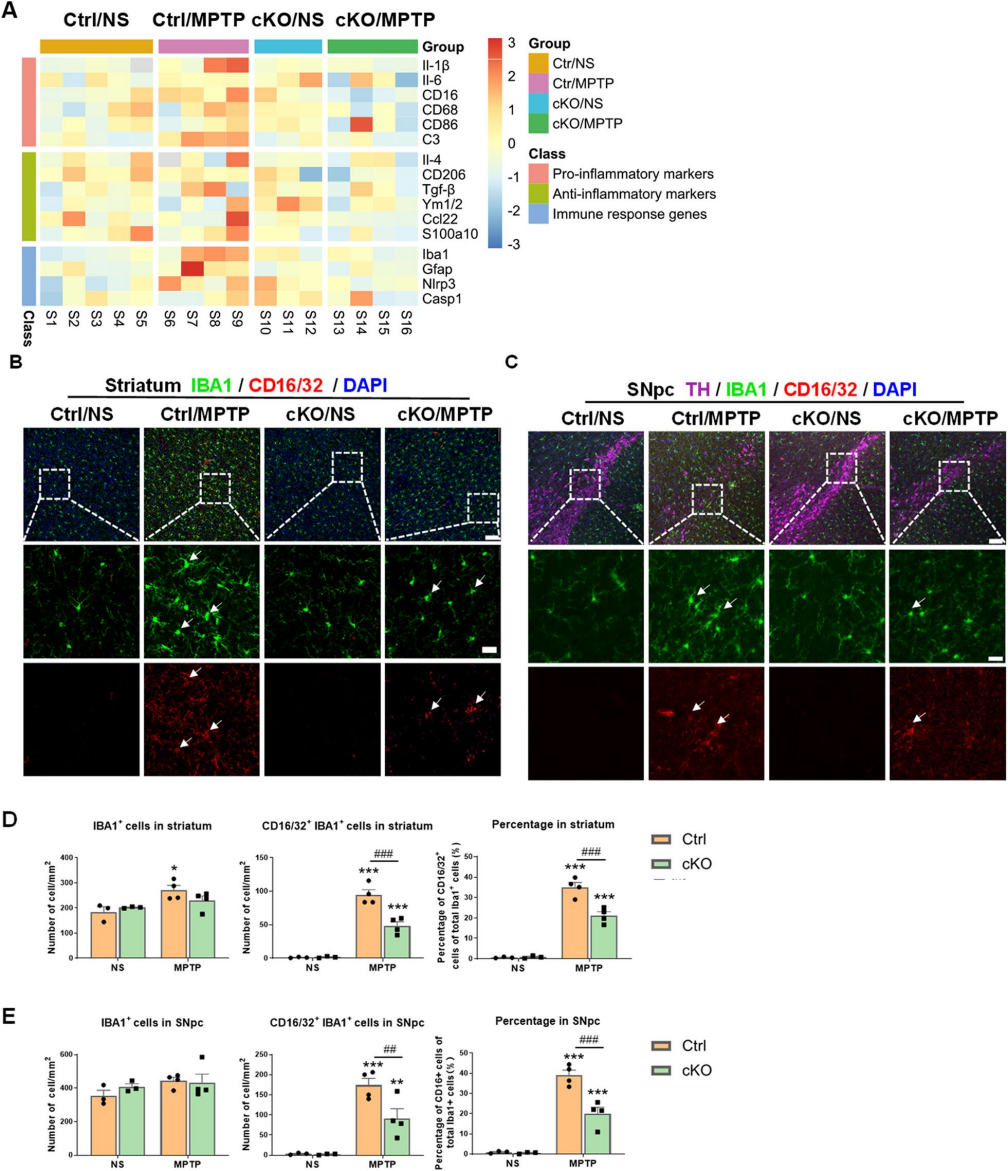

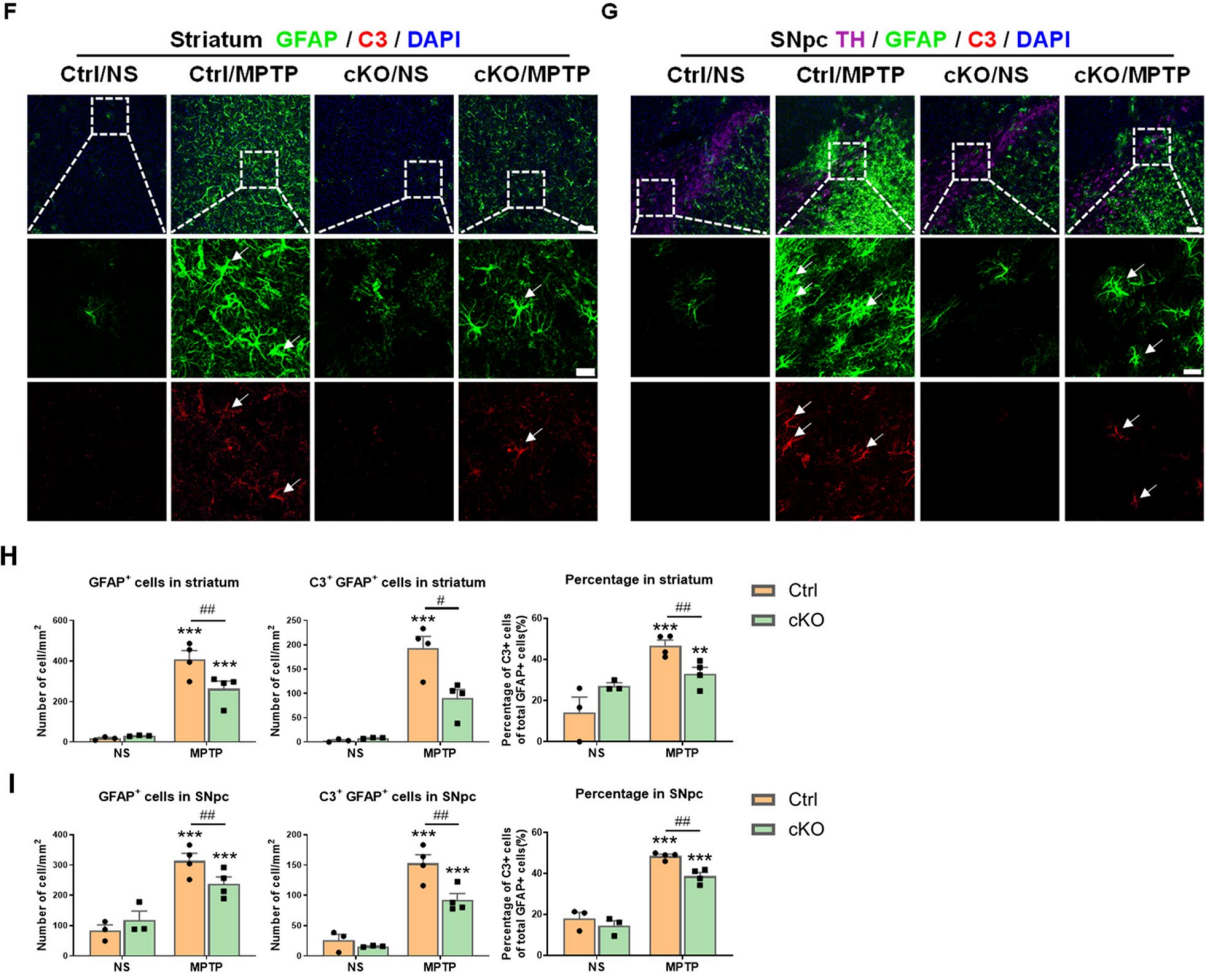
Glial polarization and activation in the nigrostriatal pathway of control and *Hif-1α* cKO mice at 7 days after MPTP administration. **A** The summary heatmap of RT-qPCR results illustrating pro- or anti-inflammatory molecules and immune response genes in the striatum of mice at 7 days after NS or MPTP injection. n = 3–5/group. **B**, **C** Assessments of microglial activation in the nigrostriatal pathway of mice. Immunofluorescence staining of IBA1 (green) and CD16/32 (red) in the striatum (**B**) and TH (magenta), IBA1 (green) and CD16/32 (red) in the substantia nigra (**C**) at 7 days after NS or MPTP administration. Scale bar, 100 µm. Small plots in the upper left quadrant showed the zoomed details. Scale bar, 20 µm. **D**, **E** Quantification of the number of IBA1^+^ and IBA1^+^ CD16/32^+^ cells (left panel) and the percentage of CD16/32^+^ cells of total IBA1^+^ cells (right panel) in the dorsal striatum and SNpc of mice. n = 3–4/group. **F**, **G** Assessments of astrocyte activation in the nigrostriatal pathway of mice. Immunofluorescence staining of GFAP (green) and C3 (red) in the striatum (**F**) and TH (magenta), GFAP (green) and C3 (red) in the substantia nigra (**G**) at 7 days after NS or MPTP administration. Scale bar, 100 μm. Small plots in the upper left quadrant showed the zoomed details. Scale bar, 20 µm. **H**, **I** Quantification of the number of GFAP^+^ and GFAP^+^ C3^+^ cells (left panel) and the percentage of C3^+^ cells of total GFAP^+^ cells (right panel) in the dorsal striatum and SNpc of mice. n = 3–4/group. Differences were analyzed by two-way ANOVA followed by LSD multiple comparison tests. *p < 0.05, **p < 0.01, ***p < 0.001 vs normal saline (NS) control. ^#^*p* < 0.05, ^##^*p* < 0.01, ^###^*p* < 0.001 vs Cre-negative control

Since blunt innate immune response was observed after conditional deletion of *Hif-1α* in microglia, we further established the MPTP-induced PD model in Cre-negative control and *Hif-1α* cKO mice to explored whether there were differences in microglial phenotypes and functions between the two genotypes of mice. The transcriptional levels of pro-inflammatory, anti-inflammatory and immune response genes in mouse striatum were examined by RT–qPCR. In general, after the treatment of MPTP, deficiency of HIF-1α in microglia resulted in transcriptional repression of pro-inflammatory markers such as *Il-1β, Il-6, CD16, CD68 and C3*, as well as immune response genes including *Iba1, Gfap* and *Nlrp3.* In spite of MPTP administration, there was no significant difference in the transcriptional levels of anti-inflammatory markers between Ctrl and cKO mice, except for *Ym1/2*. Interestingly, specific knockout of *Hif-1α* in microglia prompted the expression of *Ym1/2* and *Nlrp3* in NS-treated cKO mice, in comparison to NS-treated Ctrl mice (Fig. 8A, Additional file 1: Figure S6). The summary results suggested that HIF-1α deficiency in microglia was accompanied by the reduction of neuroinflammatory responses induced by MPTP administration. Western blotting was performed to assess the protein levels of GFAP and IBA1 in the mouse striatum 7 days after exposure to MPTP. The results revealed that MPTP administration elevated levels of GFAP protein both in Ctrl and cKO mice, while the levels of IBA1 remained unaltered (Additional file 1: Figure S7). Immunofluorescent staining was conducted to further investigate the microglial activation states. The numbers of microglia in the nigrostriatal pathway tended to return to the normal levels 7 days post-MPTP injection with the exception of microglia in the striatum of Ctrl mice. Both the quantity and percentage of CD16/32^+^ microglia were increased regardless of the genotypes. However, they reduced dramatically in cKO PD mice compared to Ctrl PD mice (Fig. 8B–E). Further, the neurotoxic activation of astrocytes was evaluated by immunofluorescent staining using a complement C3 antibody. In MPTP-induced PD mice, the numbers of GFAP^+^ astrocytes and C3^+^ astrocytes in the nigrostriatal pathway were significantly increased. However, the density and percentage of C3^+^ astrocytes in cKO mice were dramatically lower than those in Ctrl mice after the treatment of MPTP (Fig. 8F–I). These classification and quantification results revealed that lack of *Hif-1α* in microglia altered the phenotype and activation profile of microglia and astrocytes in PD mice.

### Microglia-specific deletion of Hif-1α plays a protective role in MPTP-induced PD injury

**Fig. 9:**
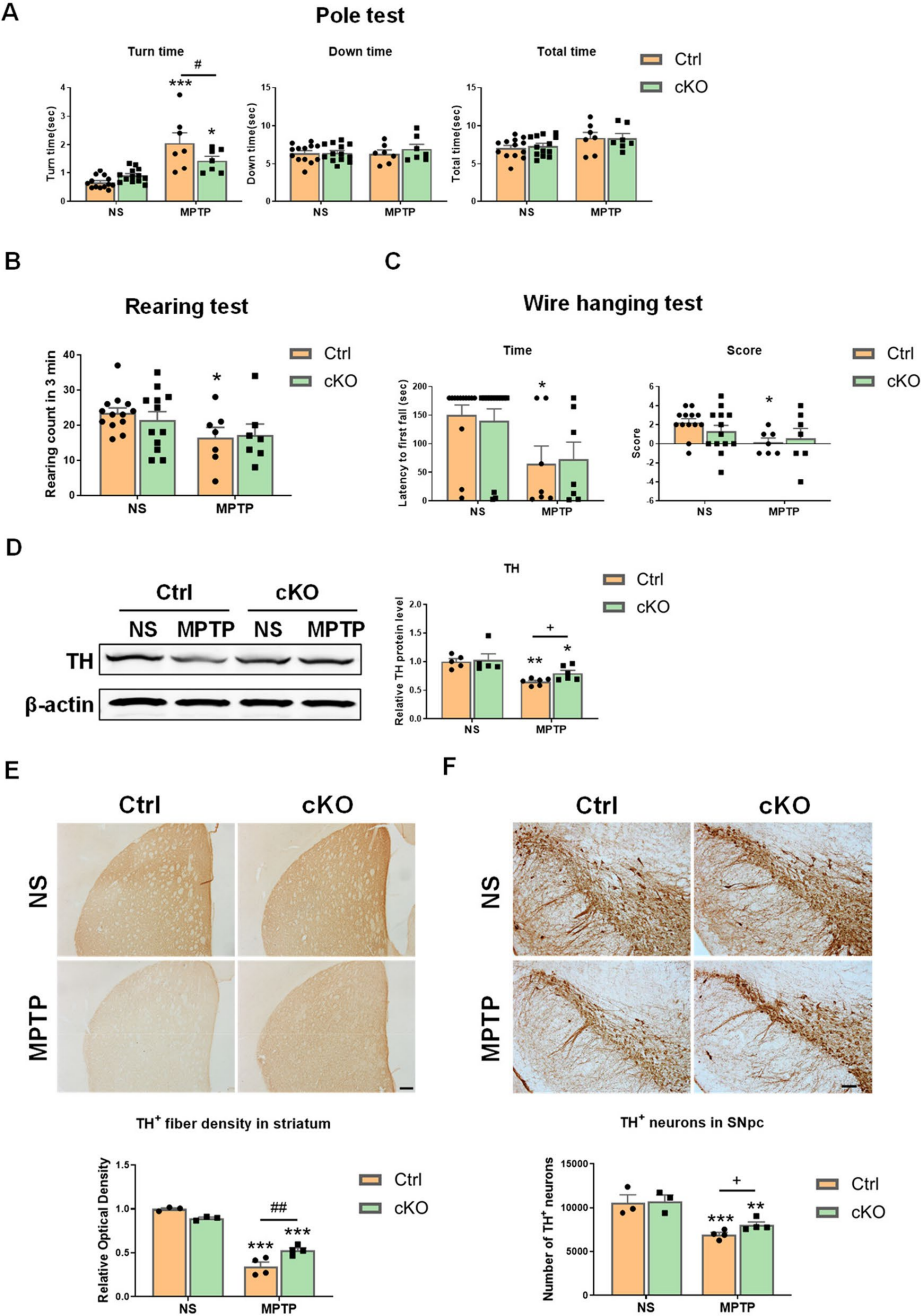
*Hif-1α* deficiency in microglia ameliorates MPTP-induced damage in the nigrostriatal pathway in mice. **A**–**C** The results of behavioral tests in mice 2 days post-MPTP administration. Time to turn around, climb down and the total time in the Pole test (**A**), the rearing counts in a 3-min period in the Rearing test (**B**), and the latency time to fall and scores in the Wire hanging test (**C**) were showed. n = 7–13 /group. **D** Protein levels of TH in mouse striatum detected by Western Blot. n = 5–6/group. The differences indicated by “+” were analyzed by unpaired t test. **E** Assessments of the optical density of TH^+^ nerve fibers in the striatum of mice by Immunohistochemistry staining. Scale bar, 200 μm. n = 3–4/group. **F** Assessment of TH^+^ neurons in the SNpc of mice by immunohistochemistry staining and stereological counting. Scale bar, 50 μm. Differences were analyzed by two-way ANOVA followed by LSD multiple comparison tests. *p < 0.05, **p < 0.01, ***p < 0.001 vs normal saline (NS) control. ^##^*p* < 0.01 vs Cre-negative control. The difference shown by “+” was analyzed by unpaired t test. ^+^*p* < 0.05

To know whether HIF-1α deficiency in microglia could alleviate PD-like pathology, we investigated behavioral changes and dopaminergic damage in the nigrostriatal pathway of MPTP-induced PD Ctrl and cKO mice. The results of behavioral tests showed that MPTP administration in Ctrl mice caused a prolonged turn time in the Pole test, a decreased rearing count in the Rearing test, and a poorer endurance on the wire and lower score in the Wire hanging test, whereas cKO mice exhibited certain resistance to such deficits (Fig. 9A–C). The dopaminergic damages in the nigrostriatal pathway were examined by western blotting, immunohistochemistry staining and stereological cell counting. MPTP exposure led to reduced protein level of TH in the striatum of Ctrl and cKO mice 7 days afterwards, but microglial deficiency of HIF-1α mitigated the decrease in TH protein levels (Fig. 9D). The optical density analysis of TH^+^ nerve fibers in the striatum and stereological counting of TH^+^ neurons in the SNpc revealed that microglia-specific deletion of *Hif-1α* induced protection of dopaminergic system against MPTP-induced injury in mice (Fig. 9E, F). Therefore, the regulation of PD-like pathology by microglial HIF-1α was observed in mice.

## Discussion

In this study, we investigated the modulatory effect of innate immune memory (IIM) in the progression of Parkinson’s disease (PD) and examined the role of HIF-1α, an innate immune regulatory molecule, in IIM and MPTP-induced PD mouse models. We conducted in vitro experiments to study the features and functional effects of IIM using BV2 microglial cells and in vivo experiments to establish mouse models of IIM. Moreover, using transcriptome analysis, we identified the differences of the enriched signaling pathways between the two forms of IIM (TR and TL). Among the related signaling molecules, we validated the regulatory role of HIF-1α in IIM. Then, we found that the existence of IIM could persist for weeks, and change the damage to dopaminergic system in the nigrostriatal pathway of MPTP-induced PD mice. Moreover, microglia-specific *Hif-1α* conditional knockout (*Hif-1α* cKO) mice failed to develop IIM. In addition, deficiency of HIF-1α in microglia partly improved the motor dysfunction and neuronal injury in PD mice. All the results indicate that HIF-1α in microglia may be a potential target for innate immunity and is involved in the regulation of pathological process of PD.

It has been widely accepted that immune system plays a pivotal role in the development, homeostasis and function of the central nervous system (CNS), with the innate immune cells and the related signaling molecules being major participants [52, 53]. As the primary innate immune cells in the brain, microglia perform immune surveillance by identifying abnormalities and triggering inflammatory responses [54, 55]. The imbalanced inflammatory environment largely contributes to the development and progression of neurodegenerative diseases, during which the highly dynamic microglia sense and converge the diverse stimulating factors [15, 56]. To date, there have been many studies about the regulators of microglia states aimed to alleviating neurodegenerative diseases.

In recent years, a growing number of studies have found the interaction between the periphery and CNS. With the proposal of ‘gut-brain axis’, ‘lung-brain axis’ and many others, the peripheral inflammation is also considered as a regulator of neural pathological progression [57–59]. However, due to the different induction methods developed to trigger inflammation in the peripheral, the consequent immune response effects in CNS are unpredictable. In our study, we utilized intraperitoneal injection of different paradigms of LPS in mice to induce distinct innate immune responses within the brain. The proposal and refinement of IIM in the brain offer a new perspective to understanding the variations in the effects of LPS on CNS [10, 13, 60, 61]. The innate immune memory property of microglia influences their immune response which is subtly influenced by their previous state each time they encounter a new stimulating factor. Consequently, the two forms of IIM (TR and TL) make IIM a new modulator of inflammatory environment in CNS.

Our experiments explored the effects of IIM in the brain elicited by peripheral administration of different sources and regimens of LPS. Although the enhanced immune response was observed in the brain of TR mice, it failed to worsen MPTP-induced dopaminergic damage in TR PD mice. Recently, Deng et al. summarized various LPS dosing regimens that may induce typical PD symptoms in mice through intraperitoneal injections [62]. However, our low-dose(s) of LPS regimen did not result in significant motor impairment symptoms. These seemingly contradictory results essentially highlighted that the source, strength and time interval of the stimulus, in addition to the dosage regimen, could alter the final specific immune effect and thus contributed differently to the progression of neurodegenerative diseases.

As mentioned above, under normal physiological conditions, the number and state of microglia are tightly regulated to maintain homeostasis. However, in the presence of disruptive factors such as injuries and diseases, the dysregulated microglia proliferate and adopt altered molecular signatures [23, 24, 63]. In previous studies, researchers have explored various markers expressed by microglia to better describe and differentiate their phenotypes and functions under different conditions [64, 65]. In our study, we utilized CD16/32 as an indicator of the pro-inflammatory phenotype in microglia. It was found that a few weeks after the first stimulus (1x or 4xLPS), the density of microglia in both groups restored to normal level regardless of the sustained expression of CD16/32, which indicated that the restoration of microglial homeostasis was not solely accompanied by the quantity recovery. At the same time, although there was no significant difference in density or proportion of CD16/32^+^ microglia between the two groups, the second stimulus did induce different inflammatory responses. These results revised our previous understanding of microglial homeostasis and revealed the limitations of the simplified classifications in defining microglial states. In recent years, a growing number of academics have proposed a multidimensional perspective that integrates various fields, such as epigenetics, metabolomics, transcriptomics, and proteomics to characterize the states of microglia [66].

Human beings are exposed to a variety of pathogens throughout their lives, which means the states of microglia in the brain are closely related to their environment. A wide range of modifications of microglia thus result in unique CNS immune status among individuals. Meanwhile, the course of many chronic neurodegenerative diseases, such as Alzheimer's disease and Parkinson’s disease, is progressive, occurring without a precise time point. Previous studies have mostly focused on the exact impact of microglia under specific experimental conditions. Works on microglial IIM, including this study, suggest more finely that we should take a retrospective view of the microglia state over the long term. Recently, it has reported that regulating the expression of GSDMD in peripheral myeloid cells can affect microglial innate immune training and neuroinflammation in brain, thereby influencing the disease progression in h-α-synuclein A30P mutant transgenic PD mice [67]. Our study, together with many other researches, revealed the regulatory role of IIM in the connection between peripheral and neurodegenerative diseases.

Since the proposal of IIM, numerous scholars have dedicated their efforts to unraveling its underlying mechanism. Epigenetic reprogramming in innate immune cells is currently recognized as the core mechanism. The external activation factors may promote epigenetic alterations within innate immune cells which result in the regulation of gene expression at both transcriptional and post-transcriptional levels. The maintenance of these epigenetic modifications is crucial for the establishment of IIM. It is worth noting that metabolic byproducts, inflammatory responses, and cytokines can induce and mediate subsequent rounds of epigenetic programs, emphasizing the importance of identifying the fundamental factors that shape phenotypic changes through intricate crosstalk [10, 13, 60, 68]. So far, molecules that affect the activation of innate immune cells, such as HIF-1α, LGALS3, SIRT1, IFN-1 and TGF-β, have been discovered and may be key players in the epigenetic modulation [26, 69–72]. Interestingly, with CD16/32 being a known pro-inflammatory marker, our study is the first to find a positive correlation between the density of HIF-1α^+^ microglia and CD16/32^+^ microglia.

Research showed that deficiency of HIF-1α in macrophages promoted metabolic reprogramming and polarization during infection [28, 73, 74]. Furthermore, myeloid-specific *Hif-1α* knockout mice lost the protective effect of β-glucan-induced IIM against S. *aureus* infection [22]. In the striatum of mice with acute innate immune memory (AIIM), the number of microglia increased sharply, and the proportion of HIF-1α^+^ microglia in TR group was higher than that in TL group. Besides, in mice with chronic innate immune memory (CIIM), the density of HIF-1α^+^ microglia in the striatum of PD mice with training memory (TR PD) was higher than that of PD mice with tolerance memory (TL PD). The results indicate that the expression of HIF-1α is different in the two forms of IIM, suggesting that HIF-1α plays a regulatory role in microglial IIM. In addition, when trying to establish AIIM models in *Hif-1α* cKO mice, we observed that the formation of IIM and the proliferation of microglia in the brain, which were caused by TR or TL stimuli, completely disappeared. This finding suggests that HIF-1α may play a crucial role in the induction and maintenance of IIM in microglia, and that the deficiency of HIF-1α in microglia leads to the dysfunction of immune response and the insensitivity to LPS.

Additionally, reactive glia cells following CNS injury exhibit heterogeneity, and their phenotypic transformation can be modulated by multiple factors [5, 75, 76]. Our results indicated that the absence of microglial HIF-1α affected the reaction of microglia and astrocytes within the nigrostriatal system of MPTP-induced PD mice. Increasing evidence has demonstrated the bidirectional communication between microglia and astrocytes, where the activated microglia can regulate the function and phenotypic conversions of astrocytes [75, 77, 78]. Our previous work had demonstrated the different dynamic change profiles of C3+ astrocytes in the striatum and the substantia nigra during the disease course in PD mice [49]. Moreover, in neurodegenerative disorders, certain molecules and cells can alter the pathological processes by inducing astrocytes to adopt a C3+ neurotoxic phenotype [77, 79]. For instance, NLY01, a GLP1R agonist, prevents the microglia-derived shift of astrocytes towards a C3+ neurotoxic phenotype in mouse model of PD, thereby mitigating the damage of dopaminergic neurons in mice [80, 81].

According to our results, due to the formation of innate immune tolerance memory, the activation of glial cells and the strength of immune response in TL PD mice (PD mice with tolerance memory) were weakened, thereby alleviating the typical pathological symptoms of PD. Mice with the absence of microglial HIF-1α could also show certain resistance to the pathological damages induced by MPTP. Therefore, regulating the intensity of immune or inflammatory response through adjusting microglia states may help to alleviate the pathology of PD. Here, microglial HIF-1α may be regarded as the core molecule of IIM, involved in regulating the pathological process of neurodegenerative diseases. It has been studied that reducing the number of immune cells can lead to an inhibited inflammatory response and restored body homeostasis [82, 83]. After the first stimulus (1x and 4xLPS), we monitored the phenotypic transitions of microglia over a span of five weeks. Interestingly, there was no significant difference in the number of microglia between the two groups throughout this time period. However, two weeks after the first stimulus, we observed significant differences in both the quantity and proportion of HIF-1α^+^ microglia between the 1x LPS group and 4x LPS group. These differences were gradually diminished over time. Additionally, it has been reported that microglial IIM can persist for several months in APP23 transgenic AD mice [12]. Thus, it remains crucial to evaluate whether there is a maximum duration for innate immune memory and whether the observed phenotypic changes involving HIF-1α in this study are associated with IIM throughout the entire duration. Furthermore, in recent years, memory characteristics of other cell types such as astrocytes and endothelial cells have also been reported. Consequently, exploring the interactions between these cells may emerge as a prominent research area in the future [84–89].

## Conclusions

**Fig. 10:**
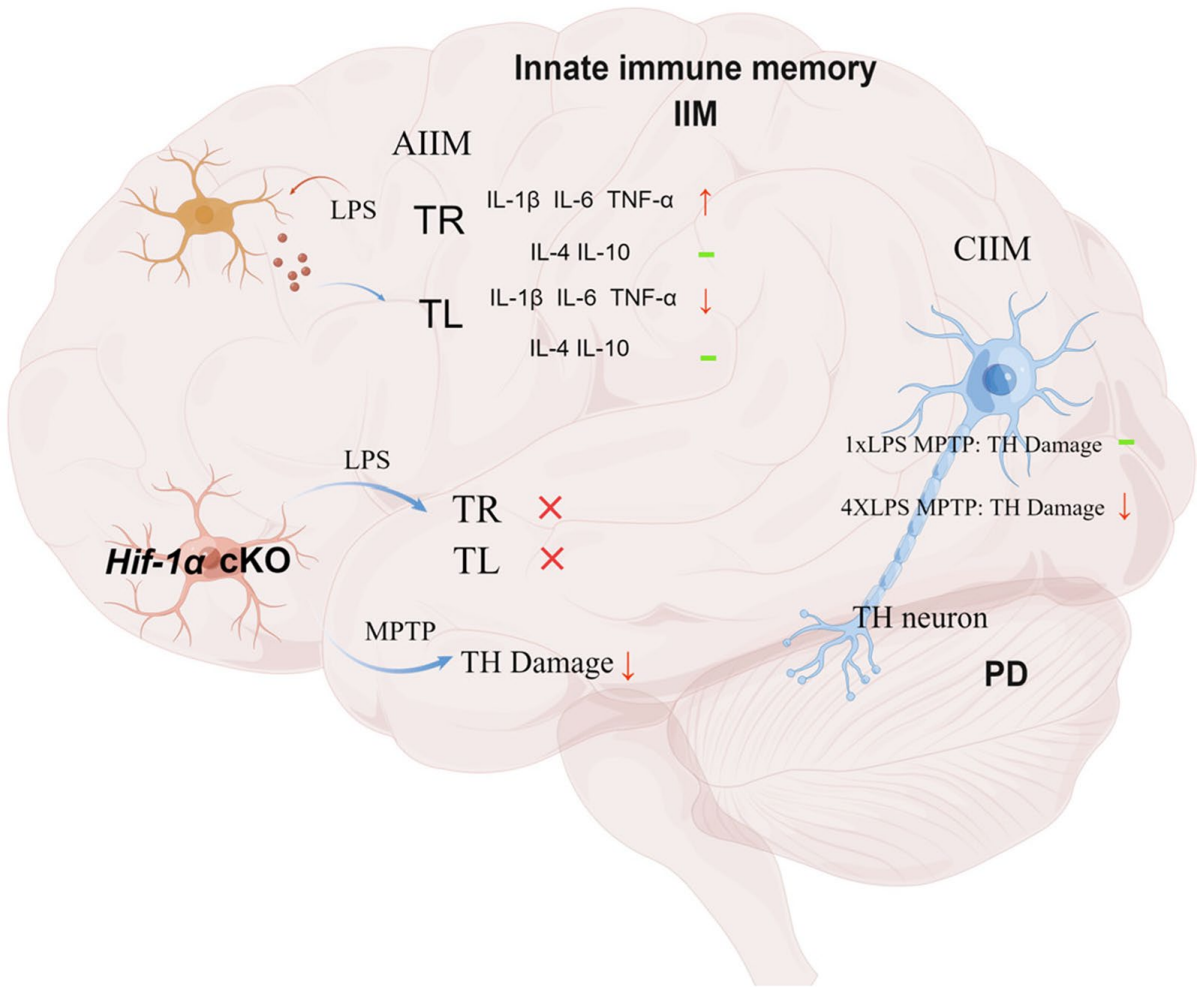
Diagram of HIF-1α in regulating the innate immune memory and the pathology of Parkinson’s disease. *AIIM* acute innate immune memory, *CIIM* chronic innate immune memory

In summary, our findings demonstrated that IIM can alter PD-like pathology in mice, with microglial HIF-1α emerging as a potential target for the regulation of IIM in Parkinson's disease. Our research expands the understanding of the pathological mechanisms of PD from the perspective of innate immune memory, and provides new insights for the intervention of this disease (Fig. 10).

### Supplementary Information

**Additional file 1: Fig. S1.** RNA-seq analysis of the striatum between mice treated with 2xLPS or 4xLPS. TR, Training; TL, tolerance. See image file. **A.** Gene ontology (GO) enrichment analysis of differential expressed genes. **B.** KEGG enrichment analysis of the enriched pathways. **Fig. S2.** Statistical graph of transcription of M1/M2-associated genes and inflammatory genes in the striatum of mice 3 h after saline or LPS treatment. See image file. n = 4–5/group. Differences were analyzed by one-way ANOVA followed by LSD multiple comparison tests. *p < 0.05, **p < 0.01, ***p < 0.001 vs control groups. #*p* < 0.05, ##*p* < 0.01, ###*p* < 0.001. **Fig. S3.** Analysis of body weight changes, Pole test results, and cytokine protein levels in Mice following LPS Injections (A, B) and MPTP Treatment (C).  See image file. **A.** The changes of body weight during and after LPS injections. n = 11–13 /group. Differences were analyzed by two-way ANOVA followed by LSD multiple comparison tests. ***p < 0.001. **B.** The Pole test results in mice 4 weeks after low-dose(s) of LPS administration. Time to turn around, climb down and total time were showed. n = 13–14 /group. **C.** Statistical graph of protein levels based on Luminex assay of pro-inflammatory cytokines (IL-1β, IL-6, TNF-α and IFN-γ), anti-inflammatory cytokines (IL-4 and IL-10) and chemokine (MCP-1) in mouse striatum 3 days after saline or MPTP treatment. n = 4 /group. Differences were analyzed by two-way ANOVA followed by LSD multiple comparison tests. *p < 0.05, **p < 0.01, ***p < 0.001. **Fig. S4.** Assessments of GFAP^+^ cells in the striatum of mice by Immunohistochemistry staining and quantification of GFAP^+^ astrocytes. See image file. Scale bar, 20 μm. n = 4/group. Differences were analyzed by two-way ANOVA followed by LSD multiple comparison tests. ***p < 0.001. **Fig. S5.** The efficiency of Hif-1α gene deletion in microglia of the Hif-1αflox/flox; Tmem119CreERT2 mice. See image file. **A.** Targeting strategy to generate *Hif-1α* ^flox/flox^ mice with *LoxP*-flanked exon 2 of mouse *Hif-1α* gene. In the presence of Cre, the *LoxP* sites were recombined and the floxed region of *Hif-1α* was deleted*.* P1 and P2, genotyping primers. **B.** *Hif-1α*^flox/flox^ mice with or without Cre transgene (Cre^+^ or Cre^−^) 2 weeks after the induction of tamoxifen were genotyped by PCR. The PCR product from the floxed allele had a PCR product of 1577 bp, whereas the knockout allele had a PCR product of 661 bp. The DNA was extracted from mouse brain. **C.** The expression of HIF-1α in microglia was assessed by IBA1/HIF-1α double-immunostaining in the brain slices of *Hif-1α*^flox/flox^ and *Hif1α*^flox/flox^; *Tmem119*^CreERT2^ mice treated with NS or 2xLPS for 3 h. Representative images of IBA1 (red) and HIF-1α (green) immunostaining in the striatum are shown. Scale bar, 25 µm. **Fig. S6.** Statistical graph of transcription of pro-inflammatory markers (*Il-1β, Il-6, CD16, CD68, CD86 and C3*) anti-inflammatory markers (*Il-4, CD206, Tgf-β, Ym1/2, Ccl22, S100a10*) and immune response genes (*Iba1, Gfap, Nlrp3, Casp1*) in the striatum of mice 7 days after NS or MPTP administration. See image file. n = 3–5/group. Differences were analyzed by two-way ANOVA followed by LSD multiple comparison tests. *p < 0.05, **p < 0.01, ***p < 0.001 vs normal saline (NS) control. #*p* < 0.05, ##*p* < 0.01, ###*p* < 0.001 vs Cre-negative control. **Fig. S7.** Protein levels of GFAP and IBA1 in mouse striatum detected by Western Blot. See image file. n = 5–6 /group. Differences were analyzed by two-way ANOVA followed by LSD multiple comparison tests. *p < 0.05, **p < 0.01, ***p < 0.001 vs normal saline (NS) control. **Table S1.** Primers used in the quantitative PCR. See Excel file.

## Data Availability

Data will be made available on request.

## Abbreviations

cKO: Conditional knockout
CM: Conditioned medium
CNS: Central nervous system
HIF-1α: Hypoxia inducible factor-1α
IIM: Innate immune memory
LPS: Lipopolysaccharide
MPTP: 1-Methyl-4-pheny1-1,2,3,6-tetrahydropyridine
NS: Normal saline
PD: Parkinson’s disease
TH: Tyrosine hydroxylase
TL: Innate immune tolerance
TR: Innate immune training
