# Supplementary material for: Hypoxia inducible factor-1α regulates microglial innate immune memory and the pathology of Parkinson’s disease
Source: J Neuroinflammation. 2024 Mar 30;21:80. doi: 10.1186/s12974-024-03070-2 (PMC10981320; doi:10.1186/s12974-024-03070-2)
Supplement: Supplementary file 1 — Additional file 1: Fig. S1. RNA-seq analysis of the striatum between mice treated with 2xLPS or 4xLPS. TR, Training; TL, tolerance. See image file. A. Gene ontology (GO) enrichment analysis of differential expressed genes. B. KEGG enrichment analysis of the enriched pathways. Fig. S2. Statistical graph of transcription of M1/M2-associated genes and inflammatory genes in the striatum of mice 3 h after saline or LPS treatment. See image file. n = 4–5/group. Differences were analyzed by one-way ANOVA followed by LSD multiple comparison tests. *p < 0.05, **p < 0.01, ***p < 0.001 vs control groups. #p < 0.05, ##p < 0.01, ###p < 0.001. Fig. S3. Analysis of body weight changes, Pole test results, and cytokine protein levels in Mice following LPS Injections (A, B) and MPTP Treatment (C). See image file. A. The changes of body weight during and after LPS injections. n = 11–13 /group. Differences were analyzed by two-way ANOVA followed by LSD multiple comparison tests. ***p < 0.001. B. The Pole test results in mice 4 weeks after low-dose(s) of LPS administration. Time to turn around, climb down and total time were showed. n = 13–14 /group. C. Statistical graph of protein levels based on Luminex assay of pro-inflammatory cytokines (IL-1β, IL-6, TNF-α and IFN-γ), anti-inflammatory cytokines (IL-4 and IL-10) and chemokine (MCP-1) in mouse striatum 3 days after saline or MPTP treatment. n = 4 /group. Differences were analyzed by two-way ANOVA followed by LSD multiple comparison tests. *p < 0.05, **p < 0.01, ***p < 0.001. Fig. S4. Assessments of GFAP+ cells in the striatum of mice by Immunohistochemistry staining and quantification of GFAP+ astrocytes. See image file. Scale bar, 20 μm. n = 4/group. Differences were analyzed by two-way ANOVA followed by LSD multiple comparison tests. ***p < 0.001. Fig. S5. The efficiency of Hif-1α gene deletion in microglia of the Hif-1αflox/flox; Tmem119CreERT2 mice. See image file. A. Targeting strategy to generate Hif-1α flox/flox mice with LoxP [file 12974_2024_3070_MOESM1_ESM.pdf]

# Supplementary materials

## Hypoxia Inducible Factor-1α regulates microglial innate immune memory and the pathology of Parkinson's disease


Hongtian Dong, Xiaoshuang Zhang, Yufei Duan, Yongtao He, Jiayin Zhao, Zishan Wang, Jinghui Wang, Qing Li, Guangchun Fan, Zhaolin Liu, Chenye Shen, Yunhe Zhang, Mei Yu, Jian Fei, Fang Huang


## Supplementary figures and legends

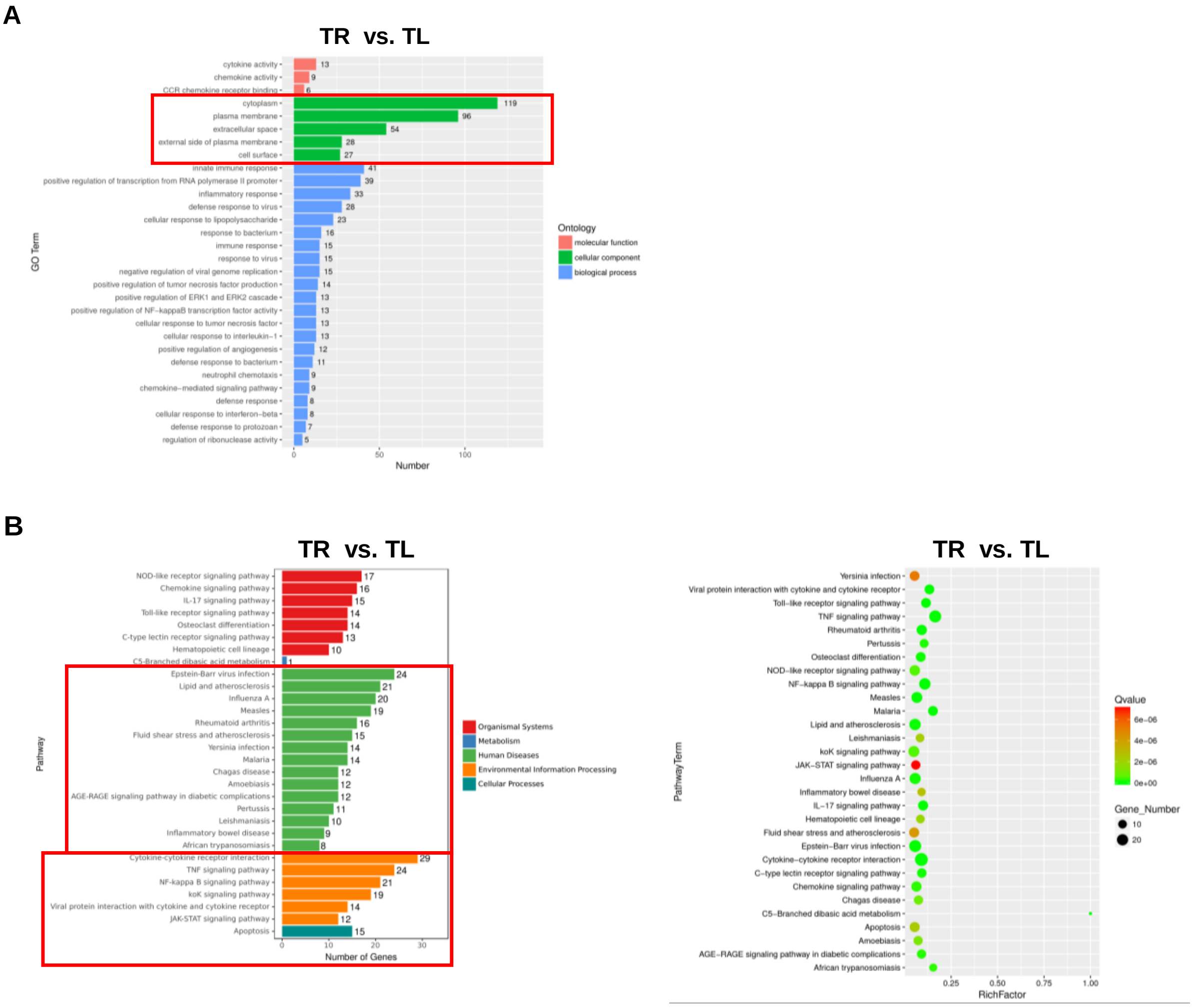

**Fig. S1. RNA-seq analysis of the striatum between mice treated with 2xLPS or 4xLPS. TR, Training; TL, tolerance.**

**A.** Gene ontology (GO) enrichment analysis of differential expressed genes. **B.** KEGG enrichment analysis of the enriched pathways.

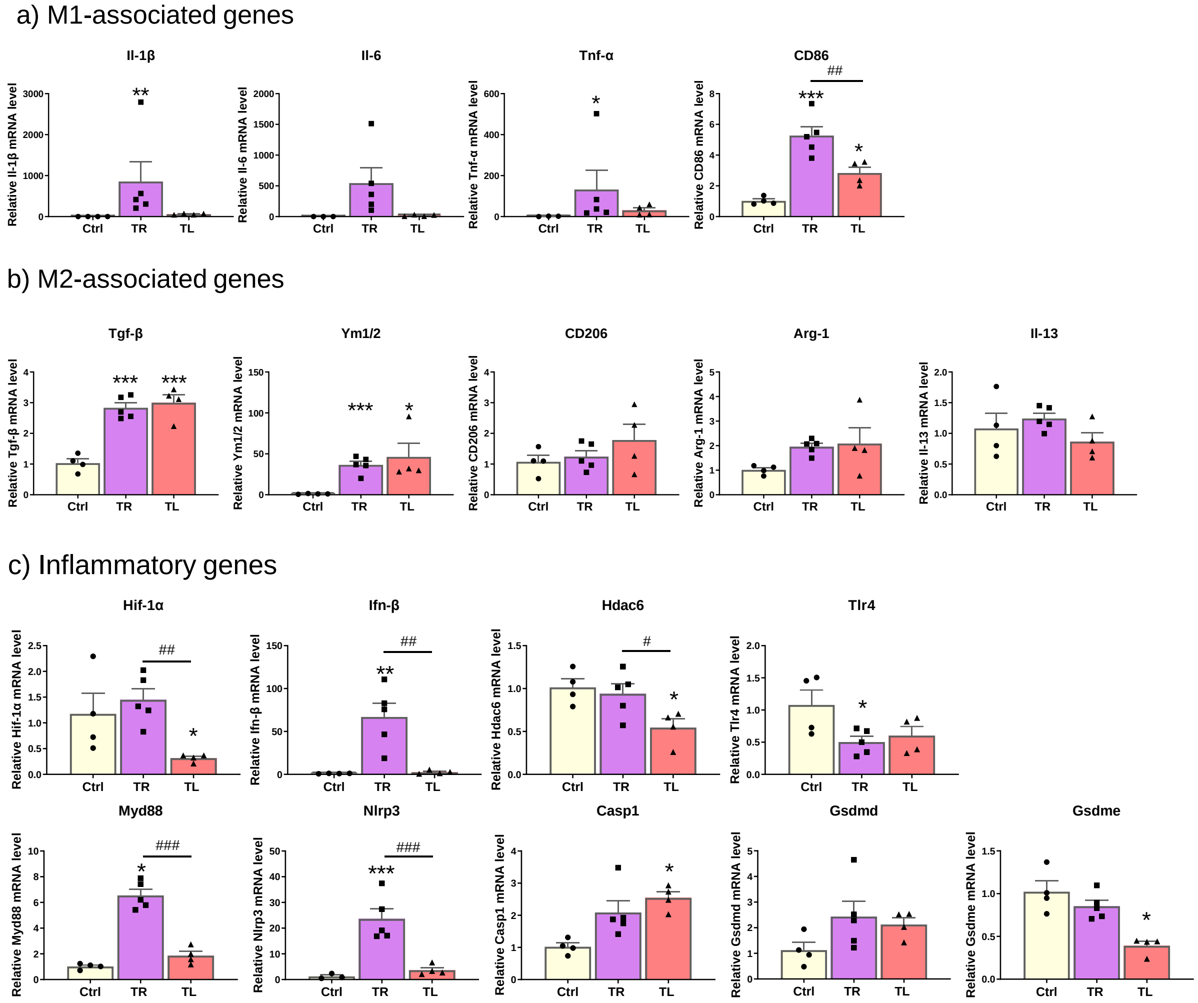


**Fig. S2. Statistical graph of transcription of M1/M2-associated genes and inflammatory genes in the striatum of mice 3 hours after saline or LPS treatment.** n = 4-5/group. Differences were analyzed by one-way ANOVA followed by LSD multiple comparison tests. *$p$ < 0.05, **$p$ < 0.01, ***$p$ < 0.001 vs control groups. #$p$ < 0.05, ##$p$ < 0.01, ###$p$ < 0.001.

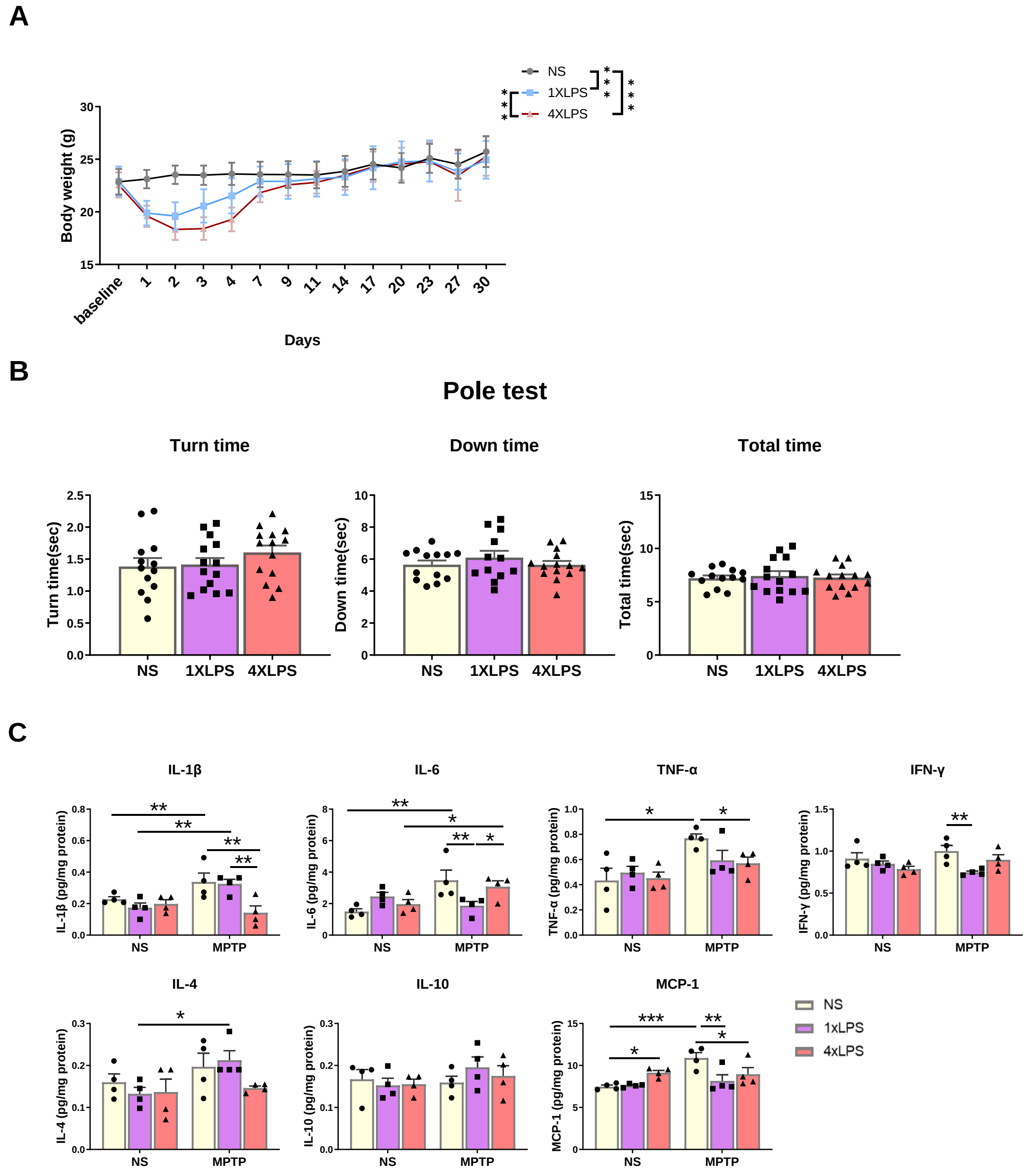


**Fig. S3. Analysis of body weight changes, Pole test results, and cytokine protein levels in Mice following LPS Injections (A, B) and MPTP Treatment (C).**

**A.** The changes of body weight during and after LPS injections. n = 11–13 /group. Differences were analyzed by two-way ANOVA followed by LSD multiple comparison tests. ***$p < 0.001$.

**B.** The Pole test results in mice 4 weeks after low-dose(s) of LPS administration.

Time to turn around, climb down and total time were showed. n = 13–14 /group.

**C.** Statistical graph of protein levels based on Luminex assay of pro-inflammatory cytokines (IL-1β, IL-6, TNF-α and IFN-γ), anti-inflammatory cytokines (IL-4 and IL-10) and chemokine (MCP-1) in mouse striatum 3 days after saline or MPTP treatment. n = 4 /group. Differences were analyzed by two-way ANOVA followed by LSD multiple comparison tests. *$p < 0.05$, **$p < 0.01$, ***$p < 0.001$.

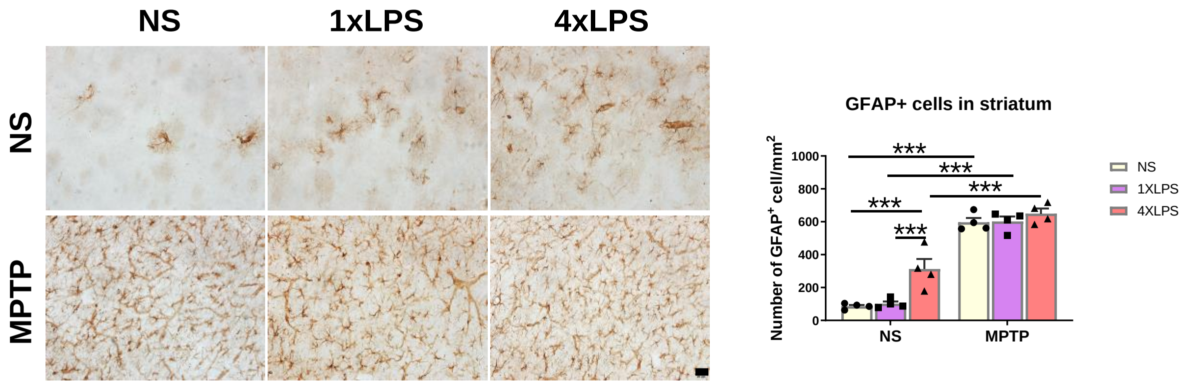


**Fig. S4. Assessments of GFAP+ cells in the striatum of mice by Immunohistochemistry staining and quantification of GFAP+ astrocytes.** Scale bar, 20 μm. n = 4/group. Differences were analyzed by two-way ANOVA followed by LSD multiple comparison tests. ***$p < 0.001$.

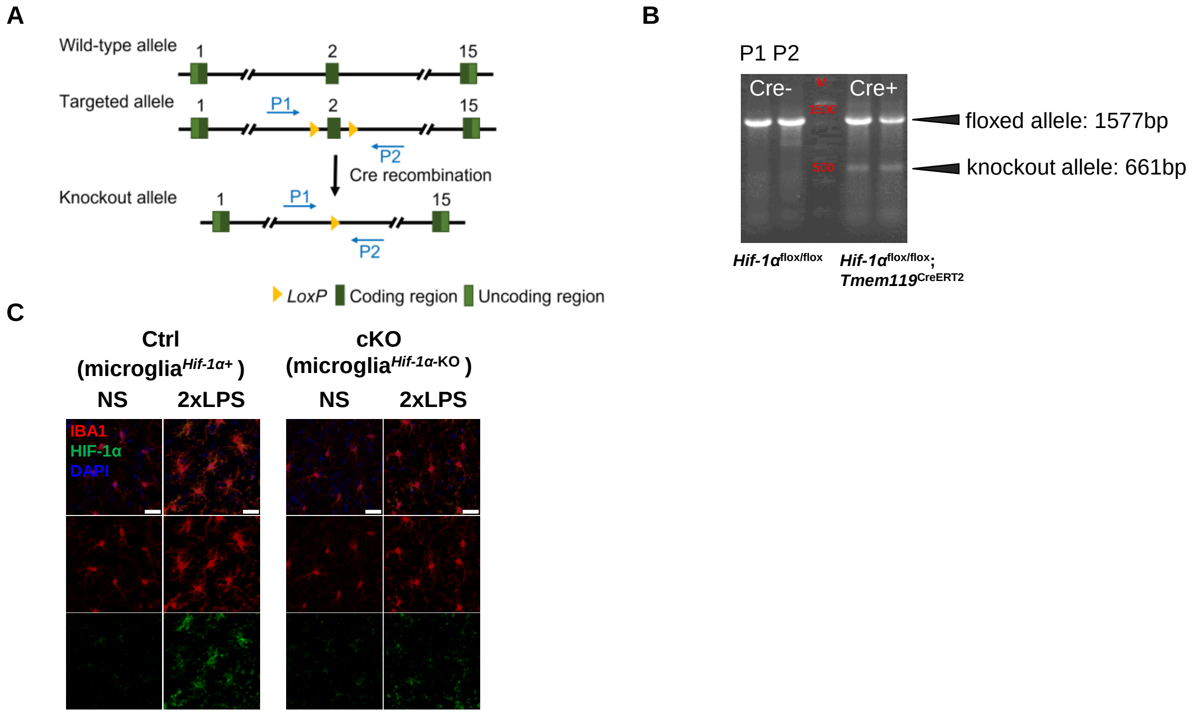


**Fig. S5. The efficiency of *Hif-1α* gene deletion in microglia of the *Hif-1α*$^{flox/flox}$; *Tmem119*$^{CreERT2}$ mice.**

**A.** Targeting strategy to generate *Hif-1α* $^{flox/flox}$ mice with *LoxP*-flanked exon 2 of mouse *Hif-1α* gene. In the presence of Cre, the *LoxP* sites were recombined and the floxed region of *Hif-1α* was deleted*.* P1 and P2, genotyping primers.

**B.** *Hif-1α*$^{flox/flox}$ mice with or without Cre transgene (Cre$^{+}$ or Cre$^{-}$) 2 weeks after the induction of tamoxifen were genotyped by PCR. The PCR product from the floxed allele had a PCR product of 1577 bp, whereas the knockout allele had a PCR product of 661 bp. The DNA was extracted from mouse brain.

**C.** The expression of HIF-1α in microglia was assessed by IBA1/HIF-1α double-immunostaining in the brain slices of *Hif-1α*$^{flox/flox}$ and *Hif1α*$^{flox/flox}$; *Tmem119*$^{CreERT2}$ mice treated with NS or 2xLPS for 3 hours. Representative images of IBA1 (red) and HIF-1α (green) immunostaining in the striatum are

shown. Scale bar, 25 μm.

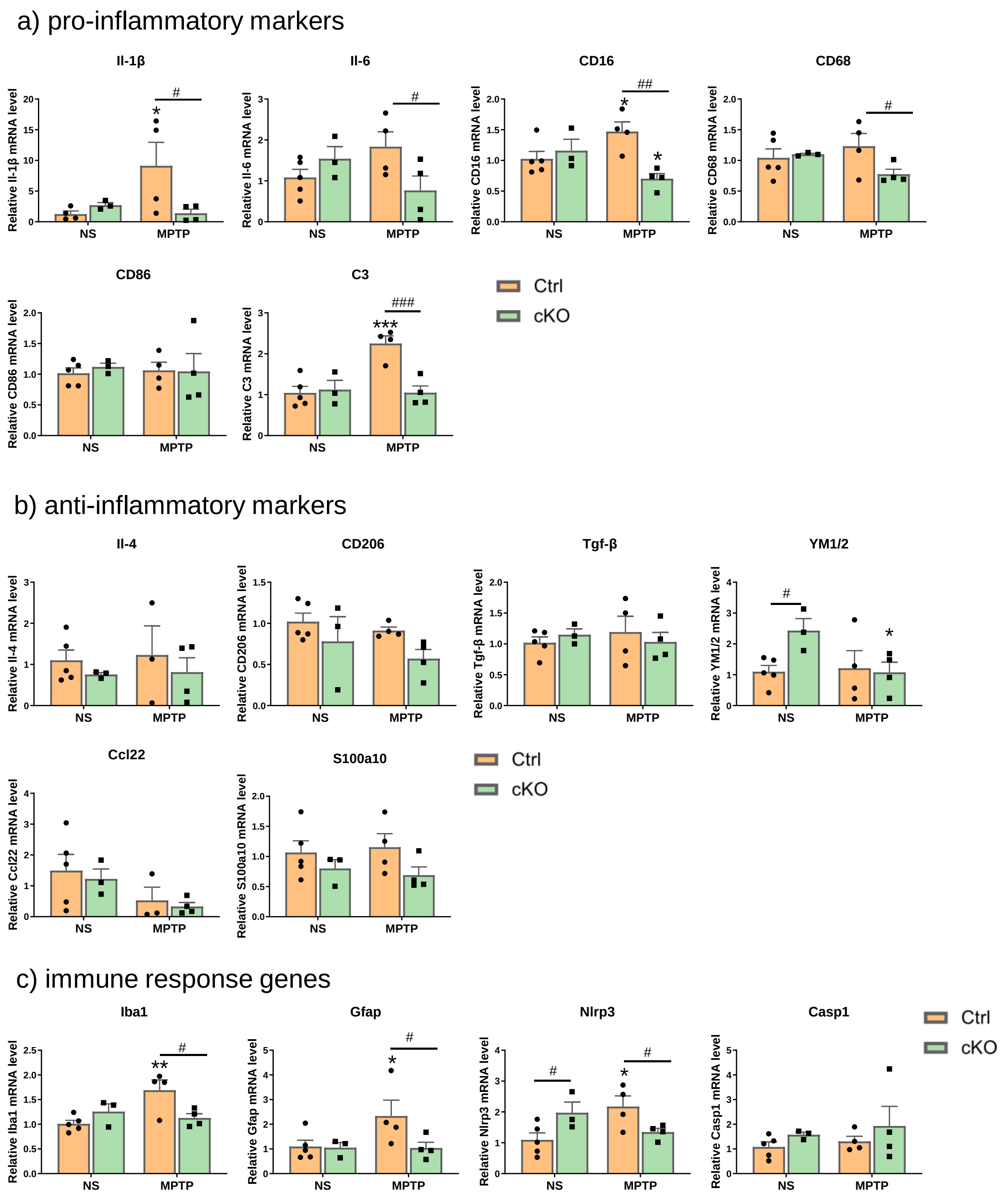


**Fig. S6. Statistical graph of transcription of pro-inflammatory markers (*Il-1β, Il-6, CD16, CD68, CD86 and C3*) anti-inflammatory markers (*Il-4, CD206, Tgf-β, Ym1/2, Ccl22, S100a10*) and immune response genes (*Iba1, Gfap, Nlrp3, Casp1*) in the striatum of mice 7 days after NS or MPTP**

**administration.** n = 3-5/group. Differences were analyzed by two-way ANOVA followed by LSD multiple comparison tests. *$p < 0.05$, **$p < 0.01$, ***$p < 0.001$ vs normal saline (NS) control. #$p < 0.05$, ##$p < 0.01$, ###$p < 0.001$ vs Cre-negative control.

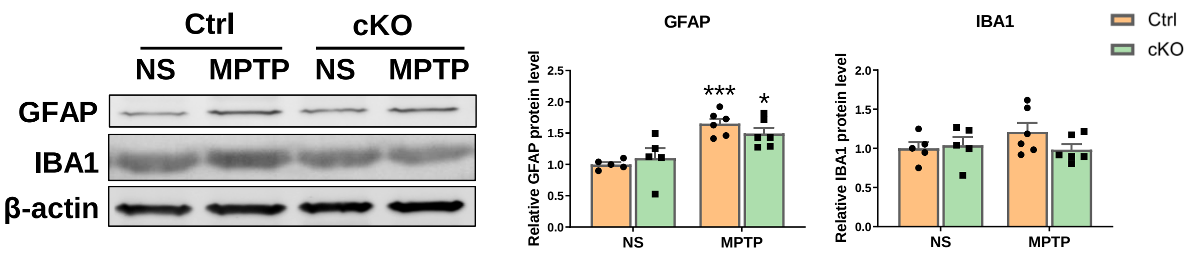


**Fig. S7.** Protein levels of GFAP and IBA1 in mouse striatum detected by Western Blot. n = 5–6 /group. Differences were analyzed by two-way ANOVA followed by LSD multiple comparison tests. *$p < 0.05$, **$p < 0.01$, ***$p < 0.001$ vs normal saline (NS) control.

## Supplementary table

### Table S1. Primers used in the quantitative PCR.

| Primers for qPCR | Sequence (5'→3') |
|---|---|
| **mArg-1-F** | AGCCAATGAAGAGCTGGCTGGT |
| **mArg-1-R** | AACTGCCAGACTGTGGTCTCCA |
| **mC3-F** | CAAAGGACCTGGAACTGCTGG |
| **mC3-R** | GGTCAGGCAGTCTTCTTCGG |
| **mCasp1-F** | AGGACATCCTTCATCCTCAGAAAC |
| **mCasp1-R** | GCAAGACGTGTACGAGTGGT |
| **mCcl22-F** | CTGATGCAGGTCCCTATGGT |

| | |
|---|---|
| **mCcl22-R** | GCAGGATTTTGAGGTCCAGA |
| **mCD16-F** | TTTGGACACCCAGATGTTTCAG |
| **mCD16-R** | GTCTTCCTTGAGCACCTGGATC |
| **mCD206-F** | ATGGATTGCCCTGAACAGCA |
| **mCD206-R** | CTCGTCAGCACCCCAGTTAG |
| **mCD68-F** | ACTTCGGGCCATGTTTCTCT |
| **mCD68-R** | GGGGCTGGTAGGTTGATTGT |
| **mCD86-F** | AGAAGCCGAATCAGCCTAGC |
| **mCD86-R** | CACTCTGCATTTGGTTTTGCTG |
| **mGfap-F** | GAGAACAACCTGGCTGCGTA |
| **mGfap-R** | CGGAGTTCTCGAACTTCCTCC |
| **mGsdmd-F** | GCTCAGTCTCCTGTCAGATGG |
| **mGsdmd-R** | GCTGGGCTGGTCCTGTAAAA |
| **mGsdme-F** | TTCCATCCCTTTGCGGTGTT |
| **mGsdme-R** | TACAGCCTGTGAGGACCAGA |
| **mHdac6-R** | CACCGCATTCAGAGGGTTCT |
| **mHdac6-R** | CCTTAAGGTGGGGCCAGAAG |
| **mHif-1α-F** | CAAGCAGCAGGAATTGGAACAT |
| **mHif-1α-R** | GCTCCGTTCCATTCTGTTCAC |
| **mIba1-F** | GGCTGGAGGGGATCAACAAG |
| **mIba1-R** | CAAACTCCATGTACTTCACCTTGA |
| **mIfn-β-F** | GTCCGAGCAGAGATCTTCAGG |
| **mIfn-β-R** | ACTACCAGTCCCAGAGTCCG |
| **mIl-10-F** | AGCCGGGAAGACAATAACTG |
| **mIl-10-R** | GGAGTCGGTTAGCAGTATGTTG |
| **mIl-13-F** | TGAGGAGCTGAGCAACATCAC |
| **mIl-13-R** | CCAGGGCTACACAGAACCCG |
| **mIl-1β-F** | CTCCATGAGCTTTGTACAAGG |
| **mIl-1β-R** | TGCTGATGTACCAGTTGGGG |

| | |
|---|---|
| **mIl-4-F** | TGCCTGGATTCATCGATAAGCTG |
| **mIl-4-R** | TTGCATGATGCTCTTTAGGCTT |
| **mIl-6-F** | CATAGCTACCTGGAGTACATGA |
| **mIl-6-R** | CATTCATATTGTCAGTTCTTCG |
| **mMyd88-F** | CCGCCTATCGCTGTTCTTGA |
| **mMyd88-R** | CTGCCAGGCATCCAACAAAC |
| **mNlrp3-F** | TGGGCAACAATGATCTTGGC |
| **mNlrp3-R** | GTACATTTCACCCAACTGTAGGC |
| **ms100a10-F** | GGTCTTCGGCACTAGCCTC |
| **ms100a10-R** | AGTCTGTCGAAACCTGGGC |
| **mTgf-β-F** | TGCGCTTGCAGAGATTAAAA |
| **mTgf-β-R** | CGTCAAAAGACAGCCACTCA |
| **mTlr4-F** | CTTGAATCCCTGCATAGAGGTAG |
| **mTlr4-R** | TGTCATCAGGGACTTTGCTGAG |
| **mTnf-α-F** | GACCCTCACACTCAGATCATCTTCT- |
| **mTnf-α-R** | CCTCCACTTGGTGGTTTGCT |
| **mYm1/2-F** | CAGGGTAATGAGTGGGTTGG |
| **mYm1/2-R** | CACGGCACCTCCTAAATTGT |
| **mβ-actin-F** | CAGGATGCAGAAGGAGATTAC |
| **mβ-actin-R** | AACGCAGCTCAGTAACAGTC |

Note: M stands for mouse
